# The Essays, Cases, Intelligence, &c.: C

**Published:** 1820

**Authors:** 


————, Mr. his account of the influenza, x. 224.

CABALA, strange opinion on the traduction of souls maintained in the, xxvii. 442.

CABANIS, P. J. G. on revolutions in and reforms of medicine, xiv. 73; on the circulation of the blood, 74; on medical education, 75; on the alliance of moral philosophy with medicine, 76; his sketch of the revolutions of medical science, xvii. 574; character of that work, xviii. 35; his account of the death of Mirabeau, xxxi. 220; his wrong treatment of that case, 223; biographical memoir of, 400; account of his works, 404.

CABBAGE, on the varieties of the, xviii. 367; how to preserve from insects, ibid.

CACHALOT, description of the, xvii. 485.

CACHEXIA AFRICANA, observations on the, ii. 33; efficacy of the Zanthoxylon in, ibid.; account of the, 171; remarks on, iii. 107; observations on its affinity to Chlorosis, xv. 143.
———— SYPHILOIDEA, observations on the, xvii. 477.
CACHEXY, observations on that produced by spirituous liquors, vi. 376.
CACTUS COCCINILLIFER, analysis of the, xxvi. 342.
CADER IDRIS, in Merionethshire, supposed to have been a volcano, xix. 382; beds of porous stones found on it, 383.
CADIZ, account of the pestilential fever at, vii. 313; xx. 27, 32, 34; xxi. 454; xxxiv. 406, 499; that epidemic supposed to be the plague in a new form, xxii. 197.
CADOGAN, Dr. his method of treating children, xxvii. 117.
CÆCUM, observations on the, iii. 75; on the formation of fat in the, xxxi. 43.
CÆSALPINUS, Andr. his treatise on the circulation of the blood, ii. 369; on the nervous influence of the brain, iii. 259; acquainted with the circulation of the blood, vi. 411.
CÆSAREAN OPERATION, on the propriety of performing the, i. 309; Baudelocque on the necessity of performing it, 391; defence of the, 506; unsuccessful case of, at Manchester, ii. 74; on the fatal effects of, 231; observations on the use of the, 433; history of a fatal case, 437; opinions of the ancients concerning the, 455; vindicated, 458; farther statement of the Manchester case of, 472; explanation concerning the case, 476; a new attempt to perform, 481; observations in defence of the, iv. 168; on the necessity for the, v. 46; on superseding it, 51; unfortunate case of, 353; history of a successful case of, 584; one that was fatal, 585; another fatal case of, vi. 346; observations on cases of, 351; signs on which it should depend, ix. 77; case in which it was twice performed on the same person, xix. 555; proper when the woman has just died, xxvii. 182; fatal case of, 331; successfully performed in France, xxxiii. 162; remarks on the, 363.
CAGOTS, a remarkable race in France, alleged to be the descendants of the ancient Goths, their history, xxxiii. 211; their origin supposed to be more remote, 437; particular description of the, 441; the Goitres proceeding from the, 442.
CAIN; on the history of, xxviii. 492.
CAIRO, frequency of the stone and gravel among the inhabitants of, xvi. 164; the sarcocele prevalent at, xxv. 292.
CAIUS, Dr. his account of the sweating sickness, xx. 473.
CAJEPUT, medicinal virtues of the oil of, i. 285; in chronic inflammation of the eyes, 286; in gout and rheumatism, ibid.; head-aches, ibid.
CALAGUALA, a species of fern supposed to be a good vermifuge, xxii. 87.
CALCAGNO, Dr. on the internal use of charcoal, xxxii. 428.
CALCAREOUS EARTH, discovery of crystallized, i. 505; how analysed, vi. 374; stone supposed to have fallen from the clouds, xxi. 85.
———— SOILS, healthiness of, i. 254; seldom visited by epidemic complaints, viii. 222.
CALCINATION, that of metals, owing to combustion, i. 170; particulars of a bed of lime-stone, xxxii. 77.
CALCIS MURIAS, form of preparing the, xxxiv. 492.
CALCULI, on the removal of biliary by ether and turpentine, i. 394; on the treatment of, 499; effects of alkalis on urinary, iv. 467; an enormous one taken from the pudendum, vi. 391; experiments on urinary, 527; on the seat and physical properties of, 528; internal texture of, 529; constituent particles of, 530; on the lithic or uric acid of, ibid.; animal matter in, 534; on the classification of, vii. 60; varieties of, 61; causes of their generation, 64; in the intestinal canal of a horse, viii. 94; on the dissolvents of, ix. 242; on alkaline injections in cases of, 246; extraordinary one extracted from the fossa navicularis, 268; on the number, nature, and characters of the materials which compose, 489; great numbers found in the bladder, xi. 15; extraordinary quantity voided by a young woman, xv. 35; experimental inquiry into the nature of those of the human subject, xvi. 153; seven ingredients in, 157; on the crystallization of, 158; number of patients afflicted with, 161; rare in Ireland, 162; frequency of the disease ascribed to cyder and poor wines, 163; common in Egypt, and why, 164; claret and cyder produce the complaint, 165; on solvents for, 222; experiments with vegetable acids and alkalis, 223; on the urine of persons affected with, 224; observations on the experiments, 305; the effects of alkalies on, 309; on the solution of those of the uric kind, 501; how to treat children affected with, 503; effects of the waters of Carlsbad on, 504; case of one voided by a woman, 554; one related by Tulpius, 554; remarkable case of, xvii. 64; case of one in the urethra, xviii. 319; on the origin of, xxi. 148; analysis of some extracted from the bladder of a dog, 369; experiments on the structure of urinary, xxii. 49; on the inefficacy of solvents on, 117; on the origin of urinary, xxii. 517; effects of carbonate of potash on urinary, xxiii. 45; and of carbonate of magnesia, 477; account of one that weighed forty-four ounces, xxiv. 185; on the means of preventing the formation of, 203; quantity voided of, 525; account of a new species of urinary, xxv. 254; a large one extracted from a dog, 258; efficacy of alkaline remedies in, 261; probability of a cure for human, xxvi. 20; of the tonsils, cases of, xxvii. 503; on the efficacy of magnesia as a remedy for, xxviii. 36; xxx. 324; best mode of administering it, xxviii. 37; proper doses of it, 38; benefit of honey in, 39; case of one of long standing, 496; symptoms of, 497; on those of the gall bladder, xxix. 519; one voided from a tumour in the groin, xxx. 170; additional facts on the benefit of magnesia in, 249; xxxiv. 445; remarkable one taken from a hog, xxx. 256; cases of, 325, 326, 328, 330; on small species of urinary, xxxii. 158; an uncommon biliary one successfully treated, 169; several voided by a woman, ibid.; dumbness occasioned by, ibid.; one found in the left hypochondrium, xxxiv. 437.

CALCULOUS AFFECTIONS, efficacy of carbonate of lime in, ii. 75; frequent in Lincolnshire, viii. 224; the nettle said to be beneficial in, xii. 31; efficacy of uva ursi in, xv. 201; benefit of the carbonate of magnesia in, xxiii. 477; xxxiv. 445.

———— CONCRETIONS in the tonsils, case of, iii. 446; all quadrupeds liable to, iv. 2; effects of muriatic acid on, xv. 570; enquiry into the nature of, xvi. 153, 221, 305, 496; of the eye, xvii. 83; found in the jejunum, xxxii. 80; dumbness occasioned by, 169; remarkable ones voided by a woman, ibid.; on the tongue, xxxiv. 172; found in a tumour, 437.

CALCUTTA, ravages of small-pox at, xxix. 78; noxious exhalations at, xxx. 16.

CALDARIS, Dr. his case of artificial impregnation, i. 47.

CALDWELL, Dr. his case of an ossified fœtus and uterus, xv. 279; his oration on the subject of quarantines, xviii. 111.

CALEDON, Lord, his encouragement of vaccination, xxx. 115.

CALEDONIAN HORTICULTURAL SOCIETY, memoir of, xxviii. 134.

CALF, description of an hermaphrodite, ii. 305.

CALLANAN, Dr. his account of the influenza at Cork, x. 523.

CALLISEN, on phlegmasia dolens, vi. 55; his observations on intus susceptio, vii. 38; on emetics in apoplexy, viii. 69.

CALLITRICHE, on that genus of plants, xxix. 174.

CALLOW, Mr. his collection of medical literature, xxviii. 516.

CALMUCKS, description of their mode of living, xxv. 250; their method of making Koumiss, 251; on the physiognomy of the, xxxiii. 496.

CALOMEL, its efficacy in yellow fever, i. 78, 248; in tænia, 182; recommended in croup, iv. 67; injudiciously administered to children, 410; beneficial in the fevers of the West Indies, 568; experiments on, vii. 547; on the oxidizement of, 549; on the preparation of, 551; dangerous in cutaneous complaints, viii. 113; beneficial in hepatic complaints, 241; utility in fevers of hot climates, x. 39; liberally ministered in yellow fever, 271; a useful remedy for the diseases of children, xi. 189; its efficacy in croup, 228; on the change of its name in the pharmacopœia, xiii. 222; beneficial in yellow fever, xv. 19; on its use as a vermifuge, xvii. 453; its efficacy in phrensy, 511; its benefit in intermittent fevers, xix. 15; new method of preparing, 190; success of it in croup, 214; successfully administered in consumptive cases, 20, 219, 224, 227, 506; its effects in epilepsy, 307; process for keeping it in a constant state of powder, xix. 382; its effects in the cure of tic doloureux, xx. 181; efficacious in the cure of worms, 380; on its general uses, xxi. 118; its effects in croup, 518; on its use in liver complaints, xxii. 416, 418, 422; its benefit in dysenteric fever, 431; its efficacy in hydrothorax, xxiii. 334; on the injudicious application of, 521; its use in epidemic dysentery, xxiv. 161; on its impropriety in yellow fever, 461; effectual in the croup, 479; effects in epidemic fever, xxv. 21; prejudicial in scrofula, 62; and in hepatitis, ibid.; also in biliary calculus, 63; improper for children, ibid.; beneficial in epidemical puerperal fever, 199; its effects in obstinate vomiting, 308; process for making, 363; its efficacy in severe gonorrhœa, 445; its effects in colica pictonum, xxvi. 43; on its virtues in rheumatism, 210; its efficacy in croup, xxvii. 211; on its exhibition in typhus, 162; on its utility in dysentery, xxviii. 35, 68; in hydrothorax, 133; its effects in catalepsy, 171; recommended in typhus, 278; its effects on children, 379; not to be used alone in fever, 448; its effects in hydrophobia, xxix. 28; in catalepsy, 45; its violence dangerous in some constitutions, 211; its utility in typhus, xxx. 321; why improper in croup, xxxii. 55; yet recommended in that complaint, 80; its use in peripneumony, 363.

CALORIC, quantity of it necessary in boiling water, i. 162; sensible and latent properties of it discovered, by Black, 168; its existence denied, 505; supposed to be a fluid, iii. 188; produces heat by extension, v. 199; to be considered as a body, 551; on the prevention of its communication, xx. 97; observations on the nature of, xxii. 314; its identity with light and electricity, ibid.; on the agency of, xxv. 328; on the necessary action of, 469; on the excess of, xxvi. 89; on the increase and diminution of, xxvii. 11; combined with carbonic oxide, xxviii. 195; abstract of the different theories on, xxviii. 512; new hypothesis on, 513; on its extrication during the coagulation of the blood, xxxii. 223; augments the temperature of the blood, xxxiii. 356.

CALOTHAMNUS, description of the, xxix. 261.

CALVERT, Dr. on the use of charcoal as a substitute for cinchona, xxxiii. 155; his theory of fever, xxxiv. 158.

CALVES, infusion of the leaves of lovage, a purge for, xii. 364.

CALX MURIATA, its efficacy in scrofula, vii. 58.

CAM, Thomas, on the use of the trephine in a detachment of the dura mater, i. 328; his case of monstrosity, vii. 385.

CAMBRIDGE, Mr. his account of the influenza at Minchin Hampton, x. 203.

CAMBRIDGE, account of Addenbrooke's Hospital at, xiv. 344; regulations of that University for medical degrees, xv 295; explanation of the decree, xvi. 344; unfavourable reports concerning vaccination at, xx. 363; epidemic at, xxxiii. 429.

CAMBRIDGE,(Massachusetts,)sketch of the University and Medical School of, iv. 569; meteorological observations at, vi. 328.

CAMBRIDGESHIRE, on the intermittents in the fens of, xxi. 92; on the birds that frequent the fens of, xxvii. 380.

CAMDEN, his account of the introduction of tobacco in England, xxiv. 458.

CAMEL, observations on an eruptive disease in the, xi. 326; comparative anatomy and physiology of the, xvi. 191.

CAMELOPARDALIS, exhibited alive at Rome, xxiv. 98; description of skeletons of, 432; fine specimen of one in London, xxvi. 17.

CAMELLIA JAPONICA, instance of its flourishing in the open air, xxv. 248.

CAMERA OBSCURA, the eye a natural one, xi. 87.

CAMERON, Dr. on the virtue of coccinella in tooth-ache, v. 546; his practice of giving bark in measles, xix. 144; his case of secondary small-pox, xxiii. 123.

CAMPBELL, Dr. E. his case of hæmatemesis, with melæna, xxii. 180.

————, John, his account of the appearance of the measles in the Inverness militia, xix. 471.

CAMPER, Peter, enumeration of his works, i. 305; on the compression of the axillary artery, x. 156; his account of a remarkable tumour, xxix. 419; his method of treating the fistula in ano, xxxii. 300.

CAMPHOR, its use in gutta serena, i. 24; how discovered in vegetables, 77; on the method of obtaining, 496; efficacious in small-pox and ulcers, 502; in sphacelus, 504; beneficial in asthma, ii. 141; discovered to be a volatile oil, iii. 89; useful in dropsy of the chest, iv. 385; on the anodyne virtue of, vi. 155; its use in fevers, vii. 568; phenomenon observed in, 572; on its motion in water, 574; on the solvent properties of, viii. 49; objection to its use in puerperal fever, x. 17; serviceable in fevers of hot climates, 39; composition of the aqueous solution of, xii. 570; given in chorea, xiii. 412; obtained from peppermint, xvii. 560; its advantage in small-pox, xix. 144, *note*; in a case of dysphagia, 249; inquiry after a substitute for, xxiii. 352; how beneficial in expelling tænia, xxv. 23; its efficacy in a case of suppression of the urine, xxvi. 161; used in friction to counteract the effects of cantharides, 315; its efficacy in epilepsy, 316; used by the Chinese in eruptive diseases, xxxiii. 248.

CAMPHORIC ACID, experiments on, xxvii. 127.

CAMPION, Mr. his observations on the eye, xxvii. 257.

CAMPSIN, description of the pestilential wind so called in Egypt, xix. 115.

CANALS, on the advantages of, xiii. 117.

CANADA, vaccination introduced there, x. 546; xv. 391; observations on the climate of, xv. 446; marsh fevers prevalent in, 448; method of curing them, 449; malignant fever in, 451.

CANCER, in the lip, cured by spirit of sal ammoniac, i. 285; solution of arsenic recommended in, 309; on the matter of, 431; case of cure in a supposed one of both breasts, iv. 296; correction respecting that case, 476; cases of supposed, 545; reply of Dr. Nisbett on a case of, v. 76; on the cure of, 81; observations on an alleged case of, 188; another case of the effects of white arsenic in, 251; vi. 31; on living hydatids in, vi. 193; case of one in the cheek, 448; observations on that case, 509; case of one in the lip, 511; power of arsenic in, 512; favourable termination of one in the breast, vii. 51; observations on that of the breast, 82, 163; on the living principle in the, 83; on the structure of schirrhous, 85; on the hydatids in, 86; on the formation of cartilage in, 87; on the cysts in, 88; peculiarity of the fat in, 163; on the term, 165; less frequent in warm than cold climates, 166; on the necessity of extirpating, ibid; method of performing the operation, 167; prize question on the subject of, 300; successfully treated with arsenic, 390; on the principles to be adopted in the treatment of, viii. 295; two cases of uterine, 431; cases of, with observations, ix. 380; carbonate of lime recommended in, 381; mercury improper in, x. 49; abscess of the breast tending to, 414; extraordinary one of the breast, 448; effects of opium in, 451; extirpation the only cure for that of the uterus, xi. 34; method of performing that operation, 35; fungus hæmatodes supposed to be a species of, 210; on the progress of ulceration in, 214; account of an empiric who affected to cure, 556; observations on the treatment of, xii. 75; benefit of the atropa belladonna in, 133; the nightshade good in cases of, 136; utility of carrots in, 232; on the virtues of hemlock in, 362; cautions necessary in having recourse to operations in, 410; scrofula sometimes mistaken for, 414; tumours in the breast not always to be extirpated, 416; cancerous mammæ begin with weaning infants, 418; history of a case of open, xiii. 294; observations on it connected with histories of the disease, 361; that of the penis, 362; in the foot, 364; in the breast, ibid; of hydatids in the breast, 365; of the tongue, ibid; in the testicle, 366; in the breast, xiv. 82; in both testicles, 83; inquiry into the nature and action of, 465; observations on the infectious nature of, 466; on a predisposition to, 468; united with consumption and gout, xv. 77; its affinity to leprosy, ibid; commentaries on the treatment of, 79; pepper-stonecrop good in, 265; on the hydatid existence of, 393; that doctrine invalidated, 398, 405, 408; the effects of carbonate of iron upon, 477; on the independent life of, 478, 479; on the contamination of the glands after amputation in, 500; the animal vitality of it vindicated, xvi. 97; on the wart in, 99; on the institution for the cure of, 179; living on pure water effectual in alleviating, 383; the animalcular existence of it contested, 403; the surgical treatment of the disease benefited by the inquiry, 409; use of the preparations of iron in, xvii. 201, 325, 444; doubts concerning those cases, xviii. 16; analogy between fungus hæmatodes and, 534; xix. 430; efficacy of oil in, xix. 142; success of extirpating one of the penis, 270; on an empirical remedy for, xx. 9; benefit of opium in, 567; effects of the conium maculatum in, xxi. 11; effects of a peculiar regimen on, 508; cases of the effects of pure water on, xxii. 79, 83; sometimes hereditary, 332; case of, in the breast, 355; effect of the carbonate of iron in, 357; observations on that remedy, 447; on the independent life of, 450; case of, in the mouth, xxiii. 500; on that called the soft, xxiv. 11; on that of the eye, 12; on the aqueous treatment of, 28; on carbonate of iron in, 29; account of a practical essay on, 72; on the unguarded use of the term, 73; divided into scirrhous and carcinomatous, 74; on cutaneous, 76; on predisposition to, 77; on the treatment of, 78; early extirpation recommended, 80; of the tongue, 134; queries concerning, xxv. 117; cases of the first stage of, 275; on the effects of regimen in, 306; on the fatal termination of, xxvi. 25; lizards said to be efficacious in, 26; practical observations on, 501; Mr. Whitbread's charitable institution for, ibid; cases of, 503; practical observations on, xxvii. 67; nostrum said to be effectual for, 71; on the structure of the parts in, 366; gutta serena produced by, xxviii. 28; cure of a confirmed, 72; property of arsenic in, 141; property of iron in, 142; case of occult, ibid; use of carbonate of iron in, xxix. 34; observations on cases of, 332; case of a lip restored after, xxx. 174; case of a complicated one of the stomach, 337; that of the breast not constitutional, xxxi. 456; account of plants used in the West Indies as a remedy for, xxxii. 109; form of using them, 111; practice of Celsus in, 117; observations on that of the bladder, 157; clivers beneficial in, 255; a powder for, 524; case of one in the uterus, 339; on that of the female breast, xxxiv. 150; cases of, 152, 154; on arsenic in, 198; on a new method of treating, 384, 410, 525.

CANDLES, made from the Dutch myrtle, xiii. 37; how to place them for the preservation of sight, xiv. 13; improvement in the manufacture of, xxiii. 261; on the effects produced by the stench of, xxiv. 223; on introducing them in cases of constipation, xxxiii. 237.

CANNABINA, success of the powder of it, in tænia, xxiv. 517.

CANNANI, a surgeon in the 15th century, discovered a valve in the vena sine pari, ii. 168.

CANTERBURY, account of the hospital at, viii. 123.

CANTHARIDES, efficacy of the tincture of, in hooping-cough, i. 84; xi. 191; in asthma, ii. 169; in hydrocephalus internus, 328; recommended in hydrophobia, viii. 89; and in dropsy, xii. 470; efficacious in hydrophobia, xiii. 159; ineffectual in tetanus, xiv. 129; on the internal use of, in gleet, xv. 415; in gonorrhœa, 417; in leucorrhœa, 421; in ulcer of the bladder, 425; on its internal use in gleet and leucorrhœa, xvi. 78; practical treatise on the powers of, xvii. 279; on the administration of it in gleet, 280; on the internal use of, xviii. 22; dreadful effects of, 55; case of tetanus unsuccessfully treated with, 436; efficacious in glanders, 556; curious circumstance attending, xix. 282; serviceable in glandular swellings, 363; case of tetanus cured by, 423; efficacious in fluor albus, 528; cases of cures by the internal use of, xx. 291, 311; beneficial in diseases of the generative organs, 381, 429; and in certain female complaints, xxi. 35, 52; in seminal emissions, 519; on the advantages of, in hydrophobia, 540; on the use of, in diseases of the genitals, xxiii. 311; substitutions for, 323; xxiv. 430; their effects on the urinary organs counteracted by camphorated friction, xxvi. 315; experiments on, xxvii. 126; on the internal efficacy of, 115; beneficial in paralysis, 149; rules for the administration of, 150; on the unfavourable effects of, 452; how injurious to the bladder, xxxi. 243.

CANTHARIS, description of a new species of, i. 142; xxiii. 323; the meloe cichorei a substitute for, xxvi. 31.

CANTON, vaccination introduced there, xvi. 576; xvii. 14; xx. 490; its progress there, farther detailed, xxvi. 453.

CANVAS, method of preparing it to convey air into coal mines, xvii. 198.

CANULA, improvement in that used for tapping, xx. 262; how to construct it, 264.

CAOUTCHOUC, on the dissolution of it in ether, ii. 83; recommended in tympanites, vii. 235; description of the plants producing the, xi. 398; on the solution of, xvii. 95; on the method of dissolving it in vitriolic ether, 454; account of the process of dissolving it, xxvi. 382, 451; description of a tree that produces the, xxviii. 52, 107; method of making it into bottles, 55; on the methods of dissolving it, 56; works giving an account of, 107.

CAPPE, Dr. Robert, on the efficacy of nitrate of silver in epilepsy, i. 181; his observations on, and cases of, angina pectoris, iv. 221; on the use of argentum nitratum, 226; on the cow-pox, 429; on irregularities in the cow-pox, v. 25; his reply to Mr. Franks, 31; on the preservation of vaccine matter, 339; his case of the power of the mind in overcoming pain, xiii. 370.

CAPPEL, Dr. on typhoid pneumonia, iii. 279; on the waters of Driburg as a remedy for scurvy, iv. 85; on the Brunonian system, 367.

CAPPER, E. Pitts, his case of hiccup, vi. 17.

CAPRIMULGUS VIRGINIANS, a remarkable bird of North America, described, xxiii. 503.

CAPSICUM, its efficacy in angina maligna, v. 425.

CAPSULA, or inverting membrane of the eye, its share in producing opacity, xxxii. 70; observations on, xxxiv. 453.

CARACCAS, on the unhealthiness of the, xxvi. 10; dreadful earthquake in the, xxvii. 162.

CARBON, formation of, i. 175; a remedy for habitual costiveness, 279; on the separating it from phosphorus, 299; on the antiseptic powers of, 393; useful in hæmorrhoids, ibid; on the origin of, ii. 388; obtained by decomposing the carbonate of lime, iii. 374; quantity of it in coal, v. 468; quantity of it in the blood, vi. 192; useful in ulcers, vii. 569; given out of the blood in respiration, xi. 85; diamond proved to be pure, 86; on the excretion of it, xix. 372; on the emission of, 373; how yielded in animals and vegetables, 375; on the cold produced by the sulphuret of, xxx. 425; its use in intermittents, xxxii. 428; efficacious against mineral poison, 248, 525; proportion of it in the blood, xxxiii. 353.

CARBONACEOUS SUBSTANCES, on the combustion of, xxxiii. 121.

CARBONIC ACID, discovered by Dr. Black, i. 168; disengaged from the skin, 397; on impregnating liquids with, ii. 80; on saturating aqua ammoniæ acetatæ with, 290; quantity of it in atmospheric air, iv. 83; method of measuring that quantity, 179; in numerous odours, vi. 444; abundant in urine, vii. 475; how obtained for medicinal purposes, 569; observation on, xiii. 109; oxygen gas converted into, xix. 372; on the transpiration of, 375; injurious to fishes, xxiii. 452; preparation of, xxvi. 95; formation of, xxix. 142.

——— GAS, its effects on the atmospheric air, vi. 494; prevalent in that of marshes, vii. 120; its effects on the animal economy, ix. 230; its effects in mortification, xviii. 536; experiments on, xxii. 48.

——— OXIDE, a gaseous compound of it with caloric, xxviii. 195.

CARBONOUS GAS, its effects on the animal economy, viii. 188.

CARBUNCLE, description of a malignant one in the West Indies, xxiii. 167; method of treating it by Celsus, xxxii. 117.

CARCINOMA, account of the progress of a, xxii. 80; of the uterus, observations on, xxxii. 246, 339.

CARDAMINE, botanical description of, xviii. 273, 274.

CARDAMOM, description of that of Malabar, xxvii. 19.

CARDAN, Jerome, his exquisite sense of smelling, vi. 339.

———, Mr. his candour in a case of vaccine inoculation succeeded by small-pox, xiii. 3.

CARDIALGIA, benefit of extractum aconiti in rheumatic, iv. 184; cured by flannel, v. 520; cured by alkalies, 560; efficacy of spongia usta in, ix. 486.

CARDIFF, account of the influenza at, x. 197, 229.

CARDITIS, case of pleuritis accompanied with, xviii. 392; two fatal cases of, xxii. 252; case of, with the appearances on dissection, xxiv. 426; singular case of, xxv. 396; cases of, xxx. 151.

CARDUUS BENEDICTUS, its extract recommended in catarrhous complaints, vi. 370.

CARENDEFFEZ, Baron de, on stony concretions in the lungs, x. 327.

CARIES of the bones, on the causes of, i. 294; on amputating the extremities of the bones in cases of, x. 557; of the os unguis supervenes, 561; of the os frontis, xii. 393; of the tibia, accompanying syphilitic ulceration of the skin, xvi. 77; of the hip-joint, remarkable case of, xviii. 490; in the second cervical vertebra, xxviii. 131; on the use of caustic in cases of, xxix. 181; of the spine, xxxi. 313; how treated, ibid.

CARLISLE, Anthony, on the use of the tourniquet in restraining arterial hæmorrhages, i. 23; his account of an instrument for dividing the cornea, 332; on the indiscriminate use of caustic bougies, ii. 289; on a peculiar arrangement in the arteries of animals, 490; on an improved bistoire cache, iii. 193; his observations in favour of flexible metallic bougies, 289; on the arteries of slow moving animals, 489; his experiments in galvanism, iv. 118; his plan of a convalescent establishment, v. 305; his observations on simple fracture where union fails, vi. 201; general remarks, illustrative of those observations, 397; his explanation in reply to Mr. Wilkinson, 477; on his proposal for the cure of bubonocele, ix. 368, 396; the inventor of metallic casts for the ear, x. 171, 359; his case of strangulated hernia, xii. 337; the Croonian lecture delivered by, xv. 95; his physiological view of the circulation of the blood, xix. 191; observations on his memoir on muscular motion, xxi. 457; on monstrosity in the human species, xxxi. 338.

CARLSBAD, extraordinary effects of the mineral waters of, xvi. 504.

CARMARTHEN, account of the influenza at, x. 303.

CARMICHAEL, Richard, his case of stricture of the urethra removed by caustic bougies, xiv. 474; his account of the effects of carbonate of iron in cancer, xv. 477; on the benefits of iron in cancer, xvii. 201, 325; xxix. 34; doubts concerning his cases being genuine cancer, xviii. 16; his observations in anchylosis, xxi. 424; his work on cancer defended against the reviewers, xxii. 412, 446; observations on his opinion, xxiv. 29, 134; on the nature of scrofula, 148; remarks on his cases, xxix. 332; on the venereal complaints resembling syphilis, xxxii. 229; xxxiv. 265, 454, 513; his treatise on the venereal disease, xxxiv. 226.

CARMINATIVE, dangerous effects of Dalby's, v. 40.

CARNAUBA, a resinous tree of slow growth in South America, xxvii. 73; analysis of the wax produced by it, ibid.

CARNEIRO, Dr. his opposition to vaccination at Madeira, xxiii. 94.

CAROTIDS, on the compression of the, x. 158; case of aneurism of the, xxii. 67, 69; nervous affections cured by pressure of the, xxv. 277; xxvi. 458; observations on those of horses, xxxii. 445.

CARPUE, Mr. his galvanic experiments on the body of a malefactor, x. 383; his successful adoption of the Taliacotian process in the restoration of a lost nose, xxxiii. 160, 516.

CARRADORI, Dr. on the composition of water, i. 195; his observations on nocturnal birds of prey, ii. 79; on the respiration of frogs and fishes, ibid; his apparatus for impregnating liquids with carbonic acid, 80; his observations on the varieties of the tremella, v. 397; on glow worms, ibid; on the illumination of rotten wood, 583; his observations on camphor, vii. 573; on freeing water of its oxygen, xvii. 197; his observations on luminous animals, xxv. 513.

CARRAWAY, description of the plant, xii. 555; useful in hysterical and hypochondriacal disorders, 556.

CARRIAGE, description of one invented for the conveyance of sick and wounded, xxxi. 313.

CARRINGTON, Sir Edmund, on the propriety of preventing inoculation for the small-pox, xx. 557.

CARRON, Dr. his cases of periodic head-ache cured by bark and opium, xxvii. 410; on the use of opium in gangrene, xxxii. 430.

CARROTS, experiments to procure sugar from, iv. 347; beneficial in inflammation, v. 240; useful in cancerous and putrid sores, xii. 232; new mode of applying them with effect in ulcers and sores, xxiii. 140; xxxiii. 384.

CARSON, Dr. on the various uses of digitalis, iii. 513; on administering calomel to children, iv. 410; his evidence on the trial of Angus, xxi. 340; examination of that evidence, 343; his reply, 345; remarks on his conduct and opinions, 427, 430; xxiv. 38; vindication of, xxxii. 327.

CARTER, B. his observations on chronic and acute rheumatism, xx. 246; reply to, 356.

CARTHAGENA, in South America, epidemic fever discovered at, xx. 27.

CARTILAGE, observations on removing a, xxvii. 372; on dividing the, xxxii. 59.

CARTMELL, in Lancashire, discovery of a medicinal spring at, xxv. 84.

CARTWRIGHT, Richard, his improved method of applying caustic, v. 513.

CARUNCLES, observations on, xxxii. 156.

CARWARDINE, H. H. his observations on the action of the muscles as preventive of the reduction of luxations, xix. 288; case of dislocated shoulder, 292; his cases of tumour, xx. 483, 485; his success in reducing a dislocated shoulder, xxiv. 468.

CASAUBON, Isaac, his remarkable case, xxix. 245.

CASCARILLA, experiments on the bark of, v. 35.

CASELLI, M. his opinions respecting extraordinary heat, xviii. 579.

CASES, on the publication of medical, xiii. 207.

CASGITTI, Dr. principal physician at Athens, adopts vaccination, xii. 448.

CASSIA SENNA, analysis of the, iii. 85.

CASTOR, a Greek botanist, his garden at Rome described, xxvi. 163.

——— OIL, beneficial in cholera morbus, xii. 103; recommended by Hippocrates for obstructions and hysteric affections, xii. 470; its excellence as a purgative, xxv. 22; improvement in the administering of it, 498.

CASTS, observations on anatomical metallic, x. 357, 359.

CATALEPSY, remarkable case of, iii. 402; another of a girl, vi. 425; remarkable one of a boy, vii. 30; particulars of one in a young man, 105; another, 106; remarkable case of, with remarks on the disease, xiii. 180; transposition of the senses in, xvi. 525; attributed to witchcraft, xviii. 37; on the causes of, xxii. 486; case of periodic, xxviii. 169; observations on that case, 376; reply to the same, xxix. 45.

CATALONIA, great number of lunatics in, xxxi. 240.

CATAMENIA, case of blindness from suppressed, v. 68; instance of premature, vii. 1; costiveness produced by a check of the, xv. 102; early appearance of, in a child, xxv. 117; efficacy of rubia tinctorum in reviving suspended, xxvi. 107; case of the early appearance of, xxvii. 522; remarkable case of, at the age of nine months, xxviii. 58; another case in a girl of four years old, xxxii. 145.

CATAPLASM, benefits of one composed of mustard in inflammatory cases, xiii. 553; seeds of flax good as a, xiv. 62; a good one for malignant ulcers made of the roots of the rhodiola, xv. 71; a good one for burns, xvi. 76; an effectual one against the guinea-worm, xvii. 172; a good one in cephalalgia, 469; advantage of mustard in fevers, xviii. 369; a good one for swellings, 458; another for inflammations, 459; the melilot trefoil used for, xix. 163; a cooling one made of groundsel, 360; impropriety of warm ones in the treatment of ulcers, xxiii. 392; benefit of that of tobacco, xxv. 222; used by Galen, xxxii. 523; useful in ulcers, xxxiii. 389.

CATARACT, instrument for operating in the, i. 332; on the use of belladonna previous to operating for, iii. 367; on the use of belladonna in extracting the, vi. 352; successful treatment of, vii. 405; case of two in the same person, viii. 115; cases of successful operation for, 548; sometimes combined with amaurosis, ix. 172; case of, 173; on performing the operation with certainty, 198; case of early, x. 10; description of instruments for extracting, 65; Mr. Hey's mode of operating for the, 371; on the depression of the, 564; directions for operating in the soft, 565; on the membranous, xi. 70; on the division of the iris, xiv. 4; xvi. 1; on the causes of, 2; means of preventing the, 55; the anemone recommended in, xvii. 74; on the methods of treating the, 81; case of, 419; on the variety of operations for, xix. 101; successful case of couching in, 102; remarks on the case, 105; observations on the operation for, 310; cases of cure by depression, 312, 313; advice to operators in, xx. 174; on making an incision in the cornea, xxi. 146; sometimes a hereditary disease, 332; on the vibrating, xxii. 475; treatment of inflammation in, xxiii. 501; on the extraction of the soft, xxv 535; method of operating for it in children, xxvii. 161, 230; on the novel modes of treating, 318; on the congenital, 335; Mr. Saunders's methods of operating for the, xxviii. 257; influence of belladonna in, 258; cases of the successful extirpation of, 261, 4; instruments used for the operation, 358; origin of one of the instruments for the, xxix. 89; cases of, 93; new instruments for, 313; method of operating for, 315; cases of, with closed pupil, 317; on the different methods of treating, 324; practical observations on, 420; symptoms of, ibid; proximate cause of, 421; on the several modes of treating, ibid; on removing it by absorption, 422; case of congenital, xxx. 68; virtue of hyoscyamus in, 70; on the operation of extraction and depression in, 71; Indian method of curing, 221; operations at Greenwich Hospital, xxxi. 82; observations on the absorption of, 295; official account of the cases at Greenwich, 326; practical treatise on, 336; observations on the, xxxii. 65; causes of, ibid; formed in utero, 66; on those of new born infants, 67; on the excellence of Mr. Saunders's method of treating, 69; on extracting the hard, 73; practice of Celsus in, 117; strictures on the report of the Greenwich cases, 486; Professor Assalini's method in operating for, xxxiii. 71; observations on that practice, 192; case of, 312; on the black, 313; on the report of the Greenwich cases, 327; observations on the treatment of, 416; various textures of, ibid; method of depressing the, 417; recovery of sight by a boy born with the black, xxxiv. 85.

CATARRH, treated by a limited use of liquids, i. 20; observations on an epidemic, 340; objections to bleeding in, ii. 237; carduus benedictus recommended in that of children, vi. 370; on the treatment of, vii. 112; bleeding recommended in inflammatory, ix. 422; not to be mistaken for influenza, 460; treated with cooling liquids, diaphoretics, and purgatives, 508; observed in Gloucestershire, 515; supposed to be contagious, 517; observations on, 518; on the affinity of influenza with, x. 97; not contagious, xi. 82; observations on the progress of, 488; proximate cause of it in the atmospheric heat, 490; on the prevalence of, 557; on the utility of bark in chronic, xii. 458; on the nature and origin of, xiii. 209; propagated by contagion, 211; caught from a cat, 214; on the treatment of, 216; observed in China, 487; efficacy of the cold affusion in, xiv. 226; coughs not always indicative of, xv. 463; poppy seeds good for, xvi. 560; remedy for, xix. 73; common in Scotland, 183; cases attended with pain in the side, ibid., 187; combined with cynanche tonsillaris, 275; on the species peculiar to old persons, 276; case of, 277; one confined to the lungs, 323; assumes the appearance of influenza, 362; cases of dropsy succeeding it, 473; excited by arsenical, solution, 477; fatal cases of, xx. 468; combined with cynanche and the influenza, xxi. 259; xxii. 171; causes of its prevalence, xxi. 261, 333; saline laxative medicines effectual in, 333; on the effects of, 433; on the obstinacy of it at Edinburgh, xxii. 172, 259, 347; a general one over Europe, xxiii. 286; prevalence of chronic, 435; on the various symptoms of, xxiv. 21; historical sketches of the epidemic, 145; observations on the same, 253; its prevalence, xxv. 82, 284; that of the bladder described, xxxi. 243; benefit of cold air and water in, xxxii. 353; cases of, 354.

CATARRHAL FEVER, observations on an infectious, iv. 290; treated with emetics and opiates, 292; account of it at Manchester, ix. 522; general remarks on, 577; observed in horses, xiii. 216; dissection of a man who died of it, xviii. 36. See *Influenza*.

——— HEAD-ACHE, cured by moxa, xxxiii. 426.

CATARRHUS SUFFOCATIVUS, cases of, with dissections, vii. 387; observations on those cases, xii. 409.

CATERPILLARS, method of preserving flax from, xx. 384; found on the cratægus oxyacantha, xxi. 92; their ravages on woollen manufactures, xxvii. 376.

CATHARTICS, cases wherein they are improper for children, xii. 108; composition of an excellent one, xv. 432; their use in palsy, xxiv. 375; on their supposed virtue in gout, xxvi. 381, 481; xxxii. 383.

CATHETER, description of an improved metallic, ii. 85; on ignorance in the use of, vi. 1; method of securing it in the bladder, x. 174; on its introduction in ischuria, xiv. 451; description of an improved canulated, 490; on the force requisite for introducing it, xv. 39; improvement suggested by Mr. Cline in the, xvii. 463; on the metallic, xviii. 243; extraordinary shape produced in one, xix. 123; on passing a large one into the bladder, xxi. 314; improvement in the construction of the, xxiv. 203; on the use of large ones, xxvi. 205; successfully employed to convey liquids into the stomach, xxvii. 269; utility of the flexible elastic, xxviii. 246; observations on an improved, xxix. 190; on leaving it in the bladder, 271; superiority of the flexible gum, 233; on the use of it after lithotomy, 375; recommended immediately after that operation, xxxi. 170; account of that used by Celsus, xxxii. 299.

CATMINT (*Nepeta Cataria*,) a specific in chlorotic cases, xvii. 468; fondness of cats for it, xvii. 468; xxiv. 431.

CATS, account of an epidemic among, i. 412; on the epizootic disease among, iv. 373; case of hernia in one, v. 105; said to have been affected by the small-pox, xii. 456; influenza communicated by, 214; on the eyes of, xvii. 414; their attachment to the nepeta cataria, 468; xxiv. 31; hydrophobia produced by the bites of, xxii. 62; account of a general mortality among, xxiii. 287; fatal effects of the bite of one, 460; case of hydrophobia from the bite of one, xxv. 61; effects of charcoal on one that had been poisoned, 458; effects of poisons on, xxvi. 132, 133, 137.

CATSKILL, (North America,) account of the epidemic fever at, xiii. 304; xiv. 312.

CATTLE, contagious diseases among, xxiii. 166, 170; account of a general mortality among, 285; on contagious diseases among, 473; rules for delivering parturient, xxix. 66; poisonous effects of the meadow saffron on, xxxii. 216; severe epizootic among the horned, xxxiii. 161, 301.

CAUSTIC, a principle in vegetables, v. 364; on its application in stricture, 481, 572; improved mode of applying it, 513; in epilepsy, x. 51; new method of using it, xi. 478, 558; observations on its application, xiv. 105; its use in hydrophobia, xvi. 440; case of its use in aneurism, xvii. 437; observations on that case, xviii. 48; on its application in strictures of the urethra, 380; its virtue in ulcers, xix. 100; wrong in spasmodic affection of the urethra, xx. 437; xxii. 21; on the use and abuse of it in strictures, xxi. 79; history of the use of it in strictures, 80; on Mr. Hunter's method of applying it, 81; objections to it, 161; farther objections to it, 304; history of the practice, 375; strong censure of, 377; in what cases to be applied, 379; unreasonable prejudice against it, xxii. 22; skill necessary in the application of it, 23; remarks on cases in which it was used, 127, 136; on the merit of the common bougie, and that of the, 517; successfully applied in tetanus, xxvii. 378; its use in caries of the bones, xxix. 181; improper in fungus cerebri, xxxiii. 405.

CAUTERY, effects of the actual, in diseases of the eyes, ears, and brain, xix. 429; in ophthalmias, 430; cephalæa, 431; phrenitis, 432; malignant fevers, 434; effectual in hydrophobia, xxii. 56; used by Hippocrates, xxxii. 115; improper in fungus cerebri, xxxiii. 405.

CAVALLO, Tiberius, on the method of dissolving of caoutchouc, ii. 83.

CAVANDER, M. his substitute for coffee, iii. 270.

CAVANILLES, D. A. on the plants of Spain, i. 204.

CAVENDISH, Hon. Henry, memoir of, xxix. 397; anecdotes of, 398; his chemical discoveries, 399; his electrical observations, 406; his astronomical papers, 407.

———————, Sir Thomas, remarkable disorder in the crew commanded by, xxxi. 306.

CAVERNS, account of the most remarkable ones, ii. 492; description of lime-stone ones in America, xi. 406; fossil remains in those of Franconia, xxvii. 26; researches in those of Paris, 27.

CAYENNE PEPPER, recommended in accidents from blows of the eye, xvi. 2; successful in cure of amaurosis, xxii. 353.

CAYLEY, Dr. his observations on quackery, iv. 404; on the practice of medicine in Russia, 405; on the benefit of opium in costiveness, xv. 336; on medical reform, xvi. 347; his case of aneurism, xvii. 5; reply to, on vaccination, 240; observations on his case of aneurism, 435; xviii. 48, 374; xix. 234.

CAZAL, M. his extraordinary case of intermitting fever, xxii. 496.

CEANOTHUS AMERICANUS, description of the, xxviii. 341.

CEDAR, ashes of the, recommended in venereal complaints, iii. 504; raised from cones in England, xxvi. 473; remarkable ones, ibid.

CELANDINE, *(Chelidonum Majus,)* its efficacy in the cure of the venereal disease, xi. 139; method of administering it, 140; cases of its success, 141; botanical description of it, xvi. 558; efficacious in intermittents, 559; a remedy for warts, tetters, and the itch, ibid.

CELERY, its efficacy in relieving ptyalism, iii. 445.

CELIBACY, a duty in persons afflicted with hereditary disease, xviii. 182.

CELSUS, his method of treating tympanites, vii. 230; his description of phagedenic ulcers, viii. 419; on the qualifications of a surgeon, ix. 85; on the leprosy, 565; on the operation for the stone, xi. 3; his account of the breaking of the stone in the bladder, 19; on the membranous cataract, 70; recommended cold water in gout, 151; on the treatment of hydrophobia, xiii. 240; his use of the ligature, 392; on amputation, xiv. 103; his account of ancient empirics, 446; his account of the morbus cardiacus, xv. 379; his method of tapping for the dropsy, xx. 262; on the treatment of erysipelas, 373; on the salubrity of the shores of the Mediterranean, xxii. 107; account of his pharmacopœia, 268; on bleeding in gout, xxvi. 479; his caution on the necessity of temperance, xxviii. 217; his operation for cataract, xxxii. 69; memoirs of, 116; account of his practice, 117, 297; on impetigo, xxxiii. 134; description of the nigritias serpens, xxxiv. 232.

CELTIS AUSTRALIS, a plant growing in Southern Europe, its powerful effects in spasmodic affections, xx. 9.

CENTAURY, a substitute for gentian, xii. 221; basis of the Portland powder, ibid.; its decoction destroys lice, ibid.; botanical description of, xix. 538; beautiful blue extracted from it, 539.

CENTEGRADE, observations on the, xiv. 576.

CEPHALEA, singular case of, iv. 15; benefit of wake-robin in, xvii. 72; relieved by the actual cautery, xix. 431.

CEPHALALGIA, vervain a good remedy in, xvii. 469; efficacy of the actual cautery in cases of, xix. 431; description of a new species of, xxv. 372.

CEPHALICA SYMPATHETICA, remedy for, xvii. 562.

CEPHALICS, observations on, xxiv. 66.

CERATE, method of making an excellent, ii. 93.

CEREBELLUM, on the origin of the nerves from the, iii. 259; case of fracture, with loss of part of the, xii. 15; on matter formed in the, xviii. 78; observations on the cineritious construction of the, xxi. 158; its state in epileptics, xxxiii. 182.

CEREBRAL MATTER, observations on that of man, xxx. 55, 66.

CEREBRUM, description of an uncommon excrescence from the, xvi. 149.

CERILLO, Professor, his ointment for rheumatism, i. 385.

CERIUM, experiments on the volatility of, xxx. 426.

CERUSSA ACETATA, case of poison by, ix. 173; successful in hæmorrhage, x. 236; recommended by Hippocrates in disorders of the eyes, xii. 471; its efficacy in burns and scalds, xvii. 21; xviii. 150; its effects in hæmorrhage of the lungs, xix 8; in uterine hæmorrhage, 9, 10; in pneumonia, ibid.; in chronic menorrhagia, 11; taken by mistake, 12; on its internal use, 13; its effects in hæmatemesis, xxii. 181.

CEVALLOS, Don Peter, his observations on the inoculation of goats, x. 341.

CEYLON, disorders prevalent in the island of, ii. 4; introduction of vaccination there, xii. 383; its success, xiii. 122; xiv. 409; xvii. 517; observations on the mineralogy of, xviii. 483; state of vaccination at, xxi. 126; on the elephantiasis peculiar to that island, xxiii. 517; on the success of vaccination there, xxv. 460; account of remarkable plants in, xxvi. 30; state of vaccination at, 35, 393; on the diabetes mellitus at, 491; small-pox exterminated there, xxvii. 34; on the treatment of the dysentery at, xxviii. 93; ravages of the small-pox there, previous to the introduction of vaccination, xxix. 72, 155; description of the inoculation establishment at, 75; on the cutaneous diseases at, 164; number of persons inoculated there, xxx. 115.

CHAIN, effects of lightning on a gold, xxiv. 437.

CHALCEDONY, analysis of the blue Siberian, iv. 374.

CHALCIDE, two peculiar species of lizards described, viii. 283.

CHALDAIC LANGUAGE, on the antiquity of the, i. 438.

CHALK, salubrity of soils of, i. 254; analysis of the black, 506; useful in cancerous affections, vii, 51; experiments with, viii. 306; gas disengaged from it, by heat, 491; beneficial in cancer, ix. 381; method of preparing the medicinal mixture of, xii. 103; its efficacy in burns, xvii. 22; observations on the gouty stones of, xxii. 155.

CHALMERS, Dr. Lionel, on the causes of tetanus, xviii. 261.

————, W. on a case of diseased bladder, xvi. 251; on pulmonary inflammation, 481; answer to, xvii. 39; on navy surgeons, 158; on the treatment of pneumonia, 159; animadversions on, xviii. 56, 61.

CHALYBEATES, their efficacy in spasmodic affections, i. 50; observations on, iv. 350; their effects on the blood, viii. 174; on that of Bath, x. 184; new one discovered at Reading, xii. 240; efficacious in diseases of the chest, xiii. 23; on a carbonated one at Stow, xxii. 346; causes of their invigorating quality, xxiii. 511; discovery of one in the Isle of Wight, xxviii. 22, 72; on that of Melksham, xxx. 299, 383.

CHAMBERLAIN, Dr. Hugh, account of, xxvii. 117.

CHAMBERLAINE, William, his botanical account of the Zanthoxylon, ii. 30; on the effects of cowhage as a vermifuge, iv. 567; his discourse before the London Medical Society, v. 313; on the virtues of the yellow gum of Botany Bay, vi. 429; on medical evidence in courts of justice, vii. 283; observations on the new medicine Act, viii. 127; on the amendments in that act, x. 165, 258, 347; account of the proceedings of the apothecaries, xi. 474; on the efficacy of stizolobium, xiii. 87; case of yellow fever cured by steam of hot sugar, xiv. 185; cautions to apothecaries and vendors of medicines, xxiii. 67; account of his tyrocinium medicum, xxviii. 88, 233; his remarks on the new medicine Act, 234; on the virtues of dolichos pruriens, 413; xxix. 336; his dissertation on the duties of medical apprentices, 494.

CHAMBERS, William, on purifying the rooms of the sick, i. 245.

CHAMBON, Dr. his works on the diseases of women and children, iii. 489.

CHAMELEON, observations on the changes of its colour, xxxiii. 339.

CHAMOMILE, on the quantity of oil given by, i. 159; useful in scarlet fever, vi. 369; beneficial in puerperal fever, viii. 519; botanical description of the, xix. 458; useful in fevers, ibid.; in agues and colics, 459.

CHAMPAIGNE, replete with carbonic acid gas and tartarous acid, xvi. 162.

CHAMPIGNONS, cases of poison by, iii. 41; xv. 247; xx. 457; the species of agaricus, iii. 43; xx. 459; botanical description, 460; observations on, 566, 567; effects resulting from the domestic use of them, xxii. 503; analytical description of the varieties of, xxiii. 68; on the reproduction of, 69; sign for distinguishing the salutary from poisonous ones, ibid.; in what respects injurious, 70; remedies to counteract the poisons of them, ibid.

CHAMSERU, M. his observations on the plica polonica, xxii. 500; xxiii. 204; xxiv. 359.

CHANCRE, efficacy of alkalies in the cure of the venereal, i. 185, 504; on the poison of, vi. 183; on the dissimilar nature of gonorrhœa and, ix. 573; benefit of the sulphat of soda in, x. 499; observations on the progress and varieties of, xiii. 80; on the inutility of early application of mercury in, 395, 551; on diseases resembling the venereal, xvii. 476; case of the successful treatment with the saturnine lotion, 548; gonorrhœal matter productive of, xxii. 312; produced by inoculation, xxiii. 80; whether it is a local affection, xxvii. 142; deception in, 143; on the phagedenic, xxxi. 415; method of treating, 416; characters of the true, xxxii. 231.

CHANDLER, John, his treatise on a cold, quoted, xiv. 371.

——————, W. his case of laceration of the bladder, xv. 153; cases of hernia, 155.

CHANNING, Dr. on diseases resembling syphilis, xxix. 60; on puerperal fever, with diarrhœa, xxxii. 461.

CHANTRAN, M. his observations on mildew, iv. 372.

CHAPMAN, Dr. Isaac, his description of a new species of cantharis, i. 142; his account of the potatoe fly, xxiii. 323.

——————, John, on injuries of the head, iii. 30; on depression of the skull, 444; on a case of ptyalism removed by celery, 445; case of uterine hæmorrhage, 482.

——————, Dr. observations on his method of extracting the placenta, xi. 328.

CHAPTAL, J. A. on the effect of mordicants in dying cotton red, i. 165; on the epidermis, 244; on the acetic and acetous acids, iii. 172; discovers a new method of bleaching, iv. 469; on the air in the Mediterranean, vii. 122; his letter on vaccination, xiii. 419; his reports on nitrous fumigation against contagion, xv. 288; on manufactures prejudicial to health, xvi. 100; his experiments on colours, xxiii. 84.

CHARAMONTE, his success in the cure of ulcers, xiv. 372.

CHARCOAL, its effects on atmospheric air, i. 28; on recovering persons suffocated by, 62; its purifying property owing to a chemical action, iv. 375; recommended to correct foul air, vi. 500; useful in ulcers, vii. 569; on the inflammable air produced by, viii. 305; on its giving fixed as well as inflammable air, 307; on the decoloration of liquors by pulverized, 478; on the conducting power of, ix. 238; explosions produced by the combustion of, 371; its property in exciting galvanic influence, x. 53; additional remarks on Mr. Cruikshank's experiments with, 273; made of the birch tree, xii. 34; its effects in purifying water, 96; useful in clarifying liquors, 283; preserves meat from putrefaction, ibid.; its effects in destroying worms, ibid.; useful in herpetic exanthemata, ibid.; good for the breath, 284; its use in checking mortification, 485; made of the wood of the corylus, or hazel-nut tree, xiv. 546; effects of the fumes of, xvi. 369; fine one for drawing, xvii. 70; found in marble, xxi. 440; reciprocal action of sulphur and, 527; case of exposure to the vapour of burning, xxii. 152; fatal effects of burning, ibid.; experiments on the combustion of, xxiv. 347; decomposition of the fulminating powders by, xxv. 457; its efficacy as a styptic, 497; experiments on, xxix. 513; found in the brain, xxx. 57; alcohol sulphur obtained from red-hot, 79; proportions of sulphur and carbon, 81; its effects in cases of poison, xxxii. 248; a remedy for intermittents, 428; on its supposed efficacy in cases of poison, 524; researches on the combustion of, xxxiii. 121; identity of the diamond with, 122, 128; on its use as a substitute for cinchona, 145; announced metallization of, 339; experiments with, 476; discovery of the analogy between the diamond and, xxxiv. 209.

CHARLATANISM, on the improper sanction of, ii. 151; not more remarkable in England than abroad, xvii. 335; historical account of, xviii. 3, observations on, 286; on the history of, xxvii. 31; in Scotland, 116; remarkable instance of, 118; anecdotes of, 308, 311; modern notices of, in London, xxx. 2; new species of, xxxiii. 21.

CHARLES II. number of persons touched by him for the evil, xvii. 478.

CHARLES THE FIFTH said to have been cured by the common Germander, xvii. 467.

CHARLESTON, South Carolina, medical register kept at, x. 468; yellow fever at, xx. 27.

CHARM, remarkable one said to be effectual in the cure of epilepsy, xxxiv. 85.

CHARMEIL, M. his successful case of the Cæsarean operation, xxxiii. 162.

CHARNOCK, Richard, on purgatives in typhus, xxviii. 278.

CHATARD, Dr. his case of strangulated hernia, xiv. 206; his account of a case of fistula lachrymalis, xx. 147; observations on that case, 150.

CHATELAIN, M. his analysis of a calculous concretion, xxxii. 80.

CHATHAM, plan for preventing infectious diseases in the garrison at, i. 459.

CHAUBRY, M. on the effects of phosphorated ether in paralysis, ix. 300.

CHAUSSIER, M. on preventing the hydrophobia, ii. 469; on a new species of medicinal salt, iii. 82; the amputation of the articulating extremities, iv. 406; on the preparation of his salt, 469; his method of preserving anatomical preparations, vi. 274; on the effects of carbonous gas in the animal economy, viii. 189; on preserving animal substances, 479; his experiments on animals with hydrogenous sulphurated gas, 522; on the umbilico-mesenteric vessels, ix. 170; his experiments on the blood, xi. 47; on preserving bodies from putrefaction, 358; his memoir on puerperal fever, xxvii. 377; case of extra-uterine pregnancy, xxxii. 459; case of congenital hernia of the heart, xxxiii. 72.

CHAVASSE, Nicholas, his successful case of diabetes mellitus treated with nitric acid, iv. 211.

CHEARFULNESS, how beneficial in hot climates, xxii. 232, 464.

CHEEKS, remarkable inequality in the, xxi. 336.

CHEESE, alteration produced by air and water on, xxiv. 25; how to restore putrid, xxxiv. 254.

CHELSEA, prevalence of ophthalmia at, xxii. 260; report of diseases in the Military Asylum at, xxxiii. 418.

CHELTENHAM, account of the influenza at, x. 409; instance of imposture attempted at, xvi. 188.

——————— WATERS, method of making artificial, x. 574; analysis of the, xx. 193; xxii. 15; observations on the virtues of, xxiii. 508; diseases in which they are useful, 510; rationale of their operation, 513; on the baths at, 514.

CHELWOOD, Somersetshire, remarkable case of small-pox at, xxxiv. 225.

CHEMISTRY, applied to agriculture, i. 45; comparative view of the principal theories of, 63, 167, 269, 368; observations on animal, 53; account of the French annals of, 82; journal of, ibid; society instituted at Philadelphia for promoting, 85; sketch of revolutions in, 99; application of it to the study of botany, 72, 157, 263, 377, 495; iii. 370; v. 361, 467; aphorisms on, i. 205; on the application of pneumatic to medicine, 82, 143; new theory of, 109; on re-agents used in operations of, 265; method of employing them, 266; doubts concerning the new system of, 194; review of the principal theories of, 269; on the application of mathematics to, ii. 298; general view of the nature and objects of, iii. 189; account of Gren's principles of, 378; cyclopædia of, iv. 76; lexicon of, 178; the history of, v. 173; elements of, 175; on the discoveries of modern, vi. 413; desultory remarks on animal, 416; its utility in purifying the atmosphere, 500; account of the discovery of animal, vii. 70, 159; its influence on animal bodies, 261, 460; reply to the defence of the new system of, viii. 305; applied in the analysis of muscular fibre, 370; new nomenclature of, 380; observations on it, 550; advantages of the analytical part of, ix. 472; improved lute for the operations of, x. 192; society formed in London for promoting, xvi. 96; on the changes in the nomenclature of, xviii. 542; the philosophy of modern, 573; application of its theory to nature and art, 574; on the means and operations of, 576; the mineral kingdom developed by, xix. 206; arrangement of the substances which are the particular objects of, 574; improved state of modern, xx. 5; observations on its present state, xxi. 129; on its universal importance, xxii. 11; its defects, 83; its extensive objects require particular assiduity, 280; necessary to be studied by medical students, 305; rapid improvement of, xxiv. 36; its pre-eminence in modern science, xxvi. 18; medical advantages of, 19; extravagant application of it, 20; account of the French annals of, xxvii. 403; historical view of its rise and progress in England, xxviii. 327; discoveries in, xxix. 9; on the progress of, xxx. 9, 12; its use in the examination of the brain, 55, 68; on the equivalents in, xxxi. 167.

CHEMISTS, pocket-book for, iv. 82; their encroachments on the apothecaries, viii. 248; the old ones opposed Galen, 331; advice to modern, xvi. 104.

CHEMOSIS, a disorder of the eyes, how to treat, x. 562.

CHENEVIX, Richard, his analysis of James's powder, vi. 517; on the chemical nomenclature, vii. 239; his observations and experiments on the metallic combinations of muriatic acid, 546; his analysis of the humours of the eye, ix. 196; xi. 163; on the dissolution of raw platina, 288; on the adoption of his powder, xiii. 226.

CHENOPODIUM, recommended in nervous diseases, ix. 94; botanical description of, xii. 227; observations on its deleterious appearance, xx. 15; a remedy for worms, 20.

CHERRIES, on the fermentation of the juice of, xi. 415; fatal effects of the eating of, xxv 31; that statement called in question, 192; confirmation of it, 288; effects of eating them with the stones, 476.

CHERRY-TREE, men supported during a siege by the gum of, xvi. 166.

CHERVIL (*Chacrophyllum*), botanical description of, xii. 370; eaten by cows, 371.

CHESELDEN, his description of a peculiar species of jaundice, vi. 312; his case of umbilical hernia, vii. 357; on the operation for the stone, xi. 6; his practice defended, xxii. 225; his success in lithotomy, 339; his method of operating for the cataract, xxix. 91, 316; his character, xxxi. 344.

CHESHIRE, account of the salt works in, xxviii. 75; observations on the rock-salt of, 155.

CHESNUT-TREE, botanical description of the, xvi. 73; one said to be 1100 years old, 74; the horse-chesnut a substitute for soap, xxiv. 430.

CHEST, on cases of dropsy of the, iv. 384; treated with camphor, 385; treatment of the diseases of the, vii. 112; complaints of it in children, viii. 202; venesection useful in complaints of the, 275; pain in the, in influenza; ix. 386; case of abscess of the, xiii. 21; frequency of disorders of the, xix. 92; fatal termination of diseases of the, 370; obstinate complaint in the, relieved by digitalis, 422; elecampane good in disorders of the, 455 —*See Hydrothorax.*

CHESTER, society there for checking the progress of the small-pox, iv. 438; testimonial in favour of vaccination at, 571; account of the influenza at, x. 213; on the antiquity and salubrity of, xxviii. 308, 9; account of medical practitioners at, xxix. 374.

CHESTON, Dr. his case of a child retained in the womb fifty-two years, xxxiii. 65, 147.

CHETLEY, William, remarkable history of, xxix. 311.

CHEVALIER, Thomas, account of his lectures, v. 486; his cases of catarrhus suffocativus, vii. 387; observations on those cases, xii. 409; his description of a new forceps for extracting balls, vii. 554; his case of puncturing the dura mater, viii. 505; his observations on gun-shot wounds, xii. 174; his cases of sudden death, xxii. 158; communication of a case of supposed rabies, xxiii. 223; case of elephantiasis, xxviii. 60; observations on the bites of serpents, 156; his case of ovarian dropsy, with observations, xxx. 162; on tying diseased tonsils, 165; case of spontaneous extravasation, 167; his case of laceration of the stomach and duodenum, xxxiii. 64.

———, M. (of Berlin,) his discovery of a new varnish, xv. 391.

CHEVREUIL, M. his observations on the nitrate of lead, xxix. 517; on the new salts discovered in lead, 523; observations on his analysis, xxx. 345; on the combustion of barytes, 523; on the bitter principle of vegetables, xxxi. 519.

CHEYNE, Dr. John, on the diseases of children, ix. 89; xi. 184; on the effects of purgative medicines, xx. 180; his cases of bronchial polypi, with remarks, 471; his description of the thorax, xxvii. 353; on the history and anatomy of apoplexy, xxviii. 21; cases of apoplexy and lethargy, 211; address to, 455; adopts bleeding in those cases, xxix. 13.

CHIARENTI, Dr. on the use of the gastric juices in diseases of the stomach, ii. 467.

CHICK, queries concerning the evolution of the, v. 589; tumours found in the thorax of a, xxviii. 381.

CHICKEN-POX, description of the, v. 320; is frequently mistaken for the small-pox, vi. 487; case of vaccination suspended by, ix. 370; different names for the, xi. 432; observations on the symptoms of, xii. 455; case of its co-existence with scarlatina, 537; case of, mistaken for the small-pox, 543; medical men often deceived by, xiii. 5; various names for it in the north, 58; case of confluent, xiv. 141; supposed to constitute the spurious cow-pox, xv. 572; its prevalence at Cambridge, and the consequence, xx. 363; case of its occurrence with the cow-pox, xxvii. 171; case of confluent, mistaken for small-pox, xxxiii. 274.

CHICKENS, affected by worms, ii. 204.

CHICKWEED, a remarkable instance of the sleep of plants, xv. 264.

CHICORIUM, or wild succory, a useful remedy in cases of biliary calculi, xix. 258.

CHILBLAINS, cantharides and ammonia recommended in, xxxiii. 160.

CHILD, account of one born without limbs, ix. 63; case of spina bifida in a, x. 287; remarkable case of the death of a, xi. 461; observations on that case, 518; xii. 51, 73; xiii. 515; should not suck too often, xiii. 30; singular conformation of the urinary and genital organs in a female, 179; case of spina bifida in a, 496; case of mal-formation of the ureters in one, 575; on the case mentioned by Academicus, xiv. 1; ascarides found in the bilious duct of a, xv. 28; non-closure of the foramen ovale in a, xvii. 78; one still-born restored to life, xviii. 307; cure of one with a large head, xxi. 293; on the attention necessary in nursing a, 294; one born without eyes, xxv. 277; uncommon large heart in one, xxx. 155; one without a gall bladder, xxxi. 50; pieces of bone discharged from the breast of one, xxxiii. 8; one retained in the womb fifty-two years, 65, 147; one born without a brain, 152; account of one with an enormous head, xxxiv. 45; another without a brain, 104; account of a piebald, 172; case of one born between the fourth and fifth month, xxxiv. 517.

CHILD-BIRTH, new theory concerning, i. 193; on the proximate cause of the fever of, ii. 387; phlebotomy improper in, 388; instance of prevention of the fever in, ibid.; abscess formed after, ii. 17; salt of tartar good in the fever of, iii. 166, 264, 363; management of difficult cases of, iv. 163; on the scriptural account of, 321; on premature delivery, v. 40; on lessening the pains in, xx. 73; proportion of deaths in, xxv. 213; case of sudden death in, xxxii. 135.

CHILD-MURDER, on giving evidence in supposed cases of, vii. 283; viii. 447; observations on the caution necessary in deciding on, ix. 74; uncertainty of the signs of, 449; the want of a foundling hospital a cause of it in Scotland, xiv. 272.

CHILDREN, flannel dresses not proper for, i. 40; on the management of, 107; method of preventing asphyxia in, 179; on the odours exhaled by, 243; on breeding them delicately, 260; those born in the spring most healthy, 261; narcotic remedies good for the eyes of, 284; on bleeding those with large heads, 390; on apoplexy in, 392; why aged persons are fond of, 494; on the thrush in, ii. 15; danger of quack medicines to, 151; cut for the stone, 287; dress recommended for, 394; on the diseases of, iv. 41; on difficult dentition in, 75, 171, 267; v. 567; efficacy of alkali in convulsive affections of, iv. 183; impropriety of giving calomel to, 410; on a peculiar tumour in, 445; danger of advertized medicines for, v. 39; on diminishing the mortality among, 183; on the physical education of, 293; on the erysipelas of new born, 511; of the poor, queries respecting the, vi. 25; on eruptions in, 234; extract of carduus benedictus good for catarrhal complaints in, 370; on promoting expectoration in, vii. 288; observations on the diseases of, 403; on the hydrocele in, viii. 34; account of an eruptive disease in, 106; on complaints after measles in, 202; essays on the diseases of, ix. 89; xi. 184; on the mortality among, ix. 358; on dentition in, x. 77; poisoned by bitter almonds, xi. 92; on the duty of preserving, 157; of the atrophia ablactatorum in, 185; on a mucous discharge common to, 453; on the management of, 466; on the clothing of, 470; danger of exposing them to cold, 473; on the disorders of, xii. 51; efficacious remedy for the diarrhœa in, 104; impropriety of cathartics for, 108; a remedy for scabby complaints in, 224; an excellent vermifuge for, xiii. 88; on the diet of, 298; on the teething of, 516; cautions respecting them at school, xiv. 374; epitome of the diseases incident to, 375; error in ascribing their disorders to worms, xv. 185; on mother's marks in, 249; an excellent cathartic for, 432; houseleek good for the thrush in, xvi. 76; frequent sufferers by calculi, 502; on the parental negligence of, xvii. 291; on the puriform ophthalmia in new born, 471; bad consequences of bringing them up hardy, xviii. 183; on some of the diseases of, 566; inhumanity practised on, xix. 240; account of an eruptive disease peculiar to, 344; characteristics of it, 345; successfully treated by women, 347; remedies for it, 348; remedy for galls in, 540; on the treatment of apthæ in, xx. 94; account of a disease in the hair of, xxi. 228; on the proper nursing of, 294; derive diseases from their parents, 329; and moral dispositions, 330; their complaints rendered unnecessarily complex, xxii. 258; mortality among them, in London, xxiii. 88; difficulty distinguishing the disease of, 128; on the couching of young, xxvii. 161; on the inflammation of the conjunctiva in, 230; impropriety of stimulating diet for, 313; advantage of vegetable regimen for, 314; on the congenital cataract in, 335; cases of early puberty in, xxviii. 58, 59; effects of calomel on, 379; on diseases incidental to dentition in, xxix. 51; process for curing the cataract in, 90; on the remittent fever of, xxx. 438; benefit of mercury in that disease, 243; number of still-born at Glasgow, xxxi. 67; caution in giving magnesia to, 341; cases of fever in them successfully treated by leeches, 363; report of diseases among those of Christ's Hospital, 479; what kind of them are most likely to live, xxxii. 334; on legitimate and illegitimate, 335; on their treatment in the school at Ackworth, 481; caution respecting supposed worm cases in, xxxiii. 290; on the cataract in, 416; on the diseases of those in the Royal Military Asylum, 418; affected by their nurses, xxxiv. 434.

CHILDREN, John George, description of his galvanic battery, xxx. 426; his experiments on the volatility of cerium, 427; account of the effects of his battery, xxxiv. 165.

CHILI, superstitious usages practised in, xiv. 439.

CHIMBORAZO, (South America,) height of that mountain, xxvi. 11.

CHIMNEYS, advice respecting the formation of, xvi. 104.

CHINA, prevalence of intermittents in, xi. 203; account of the influenza in, xiii. 487; treatise on vaccine inoculation, published in, xvi. 96; success of vaccination in, 573; xvii. 14; xviii. 29; vaccine virus conveyed thither from Manilla, xx. 488; thousands vaccinated in consequence, 489; but the practice discountenanced by the native physicians, 490; plague in, xxiii. 283; the Romans acquainted with the productions of, xxiv. 236; progress of vaccination in, xxvi. 453; on the use of tea in, xxix. 338; state of medicine in, xxxiii. 247.

CHINCOUGH, virtue of hemlock in, xii. 362; two distinct stages of, xviii. 566; on the treatment of, xix. 184, 321; general in the autumnal months, 318; resists the measles, 319; fatality of the, 366; its prevalence at Edinburgh, xxiii. 348, 433, 522; xxiv. 83; on the nature and treatment of, xxxi. 55; symptoms of, 56; description of the paroxysm, 57; cases in adults, 59; unfavourable symptoms, 61; cases of dissection, ibid.; treatment of, 65.

CHINESE, their operation of acupuncture, vii. 236; alteration of the radish of the, xii. 93; their method of healing wounds, xix. 358; their mode of raising fruit trees, xxi. 86; their practice of drinking tea, xxix. 338; their medical practice, xxxiii. 247; ignorant of the use of bread, 248.

CHING, on the vermifuge of, ii. 151; case of a child poisoned by the worm lozenges of, xvii. 173; observations on that case, 452.

CHIRAC, refutation of his theory concerning the causes of vomiting, xxx. 207; xxxii. 429.

CHISHOLM, Dr. on the anti-venereal power of the nitrous acid, i. 102; on calomel in remittents, 248; on the Cachexia Africana, ii. 171; on the temperature of Martinico, 387; on the pestilential fever of the coast of Africa and the West Indies, v. 184; on his theory of contagion, vii. 314; his opinions on yellow fever censured, xvii. 128; on the Cæsarean operation, xix. 555; on the poison of fish, xx. 461; on the infectious fever at Grenada, xxi. 241; on the use of bleeding in fevers, xxii. 459; letter to him on the classes of contagious diseases, 513; on the lues bovina, xxiii. 166, 430; his letter to Dr. Haygarth on contagion, xxiv. 22; his account of a contagious disease in the West Indies, 166; on the contagious influence of dead bodies, 422; on the poison of fishes, xxv. 398; his case of ruptured spleen and liver, xxvi. 337; on bleeding in typhus, xxviii. 476; on diseases attributed to mercurial action, 508; on giving calomel in fever, xxix. 213; on the beneficial effects of mercury in disorders of the brain, xxxi. 497; letter from Dr. Gilpin to him on the yellow fever, xxxii. 45; his account of the guinea-worm, xxxiv. 67; on his extensive use of mercury, 325; on his doctrine of contagion, 336.

CHIZEAU, M. on the puriform distinction of the brain, iii. 70.

CHLORINE, observations on, xxvii. 340; compound of carbonic oxide with, xxviii. 195; detail of experiments to decompose, xxxi. 338; its action on different substances, xxxiii. 45.

CHLORIONIC ACID, composition of the, xxxiii. 44.

CHLORITE, analysis of the, iii. 173; chronium discovered in, xxix. 431; new detonating compound of azote with, xxx. 424.

CHLOROSIS, metallic oxyde recommended in, i. 389; bleeding improper in, v. 70; observations on, ix. 280; efficacy of the Bath waters in, x. 186; a primary disease, xv. 142; benefit of aristolochiæ in, xiv. 170; its affinity to the cachexy of the negroes, 143; observations on the treatment of, 186; catmint a specific in, xvii. 468; on Cullen's method of treating it, xxi. 31; an embarrassing disease, xxii. 46; efficacy of rubia tinctorum in, xxvi. 105.

CHLOROTIC AFFECTIONS, observations on, iii. 107, 215; on the treatment of, iv. 160; benefit of alkali in, 184.

CHOLERA MORBUS, fatal case of, ii. 155; excited by heat, iv. 299; benefit of opium externally applied in, vii. 305; treated with diluents, viii. 289; characteristics of its appearance in London, 363; causes of, 364; treated with diluents and anodynes, ibid.; on the causes of, xii. 102; remedies for, 103, 219; endemic in warm countries, 262; opiates necessary in, 263; chicken and veal broth recommended in, xvi. 381; cases of bilious vomitting in, ibid.; cases of, xviii. 287, 383; on copious evacuations in, xx. 382; is sometimes periodical, xxi. 450; cases of a mild kind of, xxii. 438; on that of infants, xxix. 51.

CHOMEL, Dr. his case of a white man whose skin became black, xxxiii. 78.

CHOPPERPOORA, a poisonous snake of India, account of the, xxix. 123.

CHOREA, singular variety of the, i. 21; cuprum ammoniacum effectual in, 22; benefit of alkalies in, v. 561; relieved by electricity, ix. 468; treated with purgatives, xiii. 179; observations on that disease, with a new theory, 401; caused by water in the brain, 406; girls more affected with it than boys, 408; pigs affected by it, 409; also dogs, 410; on the convulsions in, 411; camphor used in, 412; case of, 413; observations on a new theory of, xv. 119; may exist without hydrocephalus, 125; cases of the successful treatment of, 125; effects of zinc in, 126; cases of compound, 129; the opinion of Hippocrates on, 131; on the utility of purgatives in, 185; Dr. Coxe in vindication of his opinions on, xviii. 213; on Sydenham's theory of, 220; observations on cases of, 221; on bleeding and purging in, 223; produced by teething, 226; worms not the cause of, 227; considered by the ancients under the head of convulsions, 234; time of its appearance in young women, xix. 326; case of recovery in a child, xx. 555; remarkable case in a young woman, xxii. 246; observations on the causes of, xxiv. 476; opiate frictions recommended in, 477; efficacy of blisters in, xxv. 208, 9; cured by purgatives, xxvii. 421; cases of, in the Norwich hospital, xxix. 64; benefit of purgatives in, 65; cases of, 414; cases of periodical, xxxiii. 60; remarkable case of, in a girl, 167, 254, 431; on the internal use of nitrous argenti and the shower bath in, 273.

CHOROID, observations on the coatings of the, xxxii. 321.

CHRESTIEN, Dr. on the cow-pox, xiv. 142; on the claveau des moutons, 143; on the cure of diseases by friction, xxvi. 314; observations on his opinions, xxvii. 32, 156; on his preparation of gold, xxix. 13.

CHRISTIAN, T. on a species of vaginal hernia occurring in labour, xxx. 512.

CHRISTIANA, (Norway,) horses slaughtered for food there, xxv. 283.

CHRISTIE, J. his observations on the dysentery in the East Indies, i. 348, 461; on gun-shot wounds, ii. 442; iii. 34, 141, 143; case of puerperal convulsions, 449; reply to, 548.

——, Thomas, his letter to Dr. Saunders on hepatitis, ii. 4; his account of the progress of vaccination in Ceylon, ix. 457; his letter to the Medical Board of Bombay on the same, 537; letter to Governor North on ditto, 538; on vaccination at Jaffnapatnam, 539; xiii. 122; his account of the vaccine inoculation in Ceylon, xvii. 517; xxi. 126; on the elephantiasis of Ceylon, xxiii. 517; on vaccination, xxv. 460; on the effects of stramonium in asthma, xxvi. 160; observations on diabetes mellitus at Ceylon, 491; his account of ravages of the small-pox in Ceylon, xxix. 72, 155; on the use of blood-letting in hydrophobia, 105; on the cure of tetanus by opium and the warm bath, 415.

CHRIST'S HOSPITAL, report of the diseases occurring in boys at, xxxi. 479; annual statement respecting, xxxiii. 340.

CHROMATOMETRY, or a new method of arranging and measuring prismatic tints, xxxi. 171.

CHROMIC ACID, on some unknown combinations of it with different bases, xxxiii. 53.

CHROMIUM, quantity discovered in chlorite, xxix. 431.

CHUDLEIGH, (Cape,) on the coast of Labrador, mineralogical observations at, xxviii. 73.

CHURCH-YARDS, on the infection of, x. 177.

CHURCHES, fumigation of them recommended, xiii. 115; impropriety of burying in, 116.

CHYLE, a solution of animal and vegetable substances, ii. 80; iron particles communicated to the blood with the, iv. 186; chemical analysis of, vi. 199; how chymus is converted into, 200; its analogy with blood, 201; found in the blood after its passing through the lungs, xvi. 199; formed by the action of saliva and gastric juice, 200; on the discharge of superfluous, xvii. 509; on the composition of, xxviii. 387.

CHYLIFICATION, on the probable progress of, xvi. 274; general view of its process, 299.

CHYME, a grey coloured fluid produced by the stomach, xvi. 291; its changes in passing the ductus communis, 292; its uses, 294.

CHYMUS, chemical observations on, vi. 199.

CICADÆ AMERICANÆ, great abundance of the, vii. 319.

CICUTA, successful in cancer and asthma, ii. 169; method of using it as a plaster, iii. 170; good in rheumatism and gout, xii. 367; fatal consequences of its being mistaken for parsley, xiv. 425; botanical description of it, 426; cautions respecting it, ibid.; various appellations of, 428; its efficacy in tic doloureux, xvii. 388; xviii. 135; falsity of cures alleged to have been performed by, xxxii. 205.

CINCHONA, on the principle of tannin in, v. 34; its medicinal properties, vii. 568; beneficial in pulmonary polypi, viii. 361; efficacious in erysipelas, ix. 14; on its use in puerperal fever, x. 12; inefficacious in ague, xi. 130; on the febrifuge virtues of, 215; gelatine recommended as a substitute for, 216; its efficacy in intermittents, 247; experiments to prove that it does not contain gelatine, 266; the cortex salicis latifoliæ a substitute for, 400; xii. 373; observations on the decoction of, 564; on the febrifuge substance of, xii. 430; beneficial in rheumatism, xiv. 326; jelly a substitute for, 431; on the substitution of glue for, xviii. 34; useful in diabetes mellitus, xix. 181; its effects in acute gout and rheumatism, xix. 427; its use in ophthalmia, xxiv. 222; beneficial in scarlatina, xxv. 298; alterations in the names of, 498; on the several kinds of, xxvi. 12; used as a friction in rheumatism, 318; botanical description of the different sorts of, 325; the heart-leaved, 326; the lance-leaved, 328; the oblong leaved, 329; analysis of the, 331; medical properties of, 333; account of the Caribbean, 336; its effects in diabetes, xxxi. 146; charcoal a substitute for it in the cure of intermittents, 514; xxxii. 428; xxxiii. 145.

CINCHONIN, its influence upon the virtue of barks, xxvii. 295.

CINDER, chemical observations on finery, viii. 305; x. 273.

CINNABAR, supposed to be a composition of sulphur and mercury, viii. 573; experiments to ascertain the component parts of, xvi. 575.

CINQUE-FOIL, medicinal virtues of the, xvi. 265.

CIRENCESTER, account of the influenza at, x. 199.

CIRCULATION, on animal poisons entering the, xxix. 133; remarkable symptoms in the, xxxiv. 360.

CISTERNS, on the danger arising from leaden, xi. 464; experiments on the foul air in oil, xv. 391.

CISTUS, description of two species of the, xxv. 93.

CITIES, on the crouded state of the poor in large, iv. 396.

CITRIC ACID, analysis of the, i. 159, 302; method of preparing, 401; improvement in making, iii. 171; on the composition of the concrete, iv. 154; its general use in the fleet, v. 75; its use in medicine, vii. 568; composition of, xii. 566.

CITRUS, (the Lemon,) its medicinal uses, vii. 568.

CIVILIZATION, observations on the injury to man from, xv. 74; its imperfect state in France, xxxiii. 71.

CLANNY, Dr. his account of mineral waters in the county of Durham, xvii. 479; case of a man who vomited urine, xxxii. 41; on an annular saw, xxxiii. 191.

CLAPHAM, on supposed failures of cow-pox inoculation at, v. 86; small-pox fatal at, xiii. 251.

CLAPTON, (Middlesex,) meteorological diary kept in 1810 at, xxiii. 437; xxiv. 176.

CLARE, Lord, his extraordinary case of contused urethra, xxi. 367.

CLARET, recommended to hypochondriacs, i. 150; gout and gravel produced by, xvi. 165.

CLARGES, Sir William, his invention of a life-boat on new principles, xxi. 173.

CLARION, M. his description of a new species of phoca, viii. 282.

CLARK, Bracy, on the structure and uses of the several parts of the horse's foot, xxiii. 175; his experiments on the same, 338; his series of experiments on the foot of the living horse, xxvii. 425.

———, Dr. James, on the use of mercury in yellow fever, i. 79.

———, Dr. John, his collection of papers to promote a Fever Institution at Newcastle, viii. 562; ix. 182; on the influenza, x. 119, 208; dispute between him and Dr. Trotter, xii. 293; xiii. 133; his estimable character, xiv. 255.

———, Dr. Thomas, his description of new surgical instruments, ix. 1, 8; xii. 541, 548; his observations on the nature and cure of fever in the West and East Indies, xv. 83; on the medical department of the army, 88; on the benefit of mercury in yellow fever, xxiv. 461; on ipecacuanha enema in dysentery, xxviii. 94; his remarkable case of poison, 172; on the utility of calomel in fever, 298.

———, Mr. his case of a stone voided by a woman, with remarks on the same, xvi. 554; another of cartilaginous substances extracted from the knee-joint, xxxiii. 63.

CLARKE, Charles Mansfield, on mixtures with spermaceti, iii. 269; on female diseases, attended by discharges, xxxii. 161, 239, 336.

CLARKE, Dr. E. D. his observations on the manners of the Calmucks, xxiv. 250.

———, Dr. James, his account of vaccination at Nottingham, xv. 254; xvi. 132; a letter to, xvi. 259; his correspondence with the Jennerian Society, xvii. 369; his medical reports for Nottingham, xix. 179; xx. 467; xxi. 425; xxii. 244; xxiii. 154; xxiv. 157; xxvi. 151; xxviii. 133; on vaccination in France, xviii. 341; on a disease in dogs, xxii. 112; his case of hydrophobia, xxvii. 239; on purging in typhus, xxviii. 293; his meteorological journal at Sidmouth, xxxiii. 188.

———, Dr. J. his remarkable case of typhus, xxiii. 165.

———, Mr. the first who raised cedars of Lebanon in England, xxvi. 473.

———, Dr. John, on a case of difficult parturition, iii. 46; his communication of a case of calculous concretions, 446; on the hasty delivery of the placenta, 464; on cases in which the face presents to the os pubis, iv. 163; on the progress of vaccination, vi 95; his case of sudden death from a morbid affection of the brain, xii. 391; on an unusual case of parturition, xvi. 53; on burning the placenta, 54; his opinion on quickening, xxvii. 445.

———, Dr. Joseph, his case of arm presentation, viii. 394; his case of the amputation of the uterus, xvi. 472.

CLARKSON, Mr. his lectures on the pyramids, xxvii. 341.

CLASSIFICATION, benefits of it in medical science, xi. 566; new form of it, xx. 47; on that of physics, xxviii. 331.

CLAUBRY, Gualtier, on the use of alkalis against accidents produced by lightning, iv. 370.

CLAUDIUS, the emperor, poisoned by the agaricus deliciosus, xx. 352.

CLAVEAU, a peculiar disease among sheep, of the eruptive description, vii. 241; xii. 457; resembles the small pox, 458, 461; instances of inoculation with the, xiv. 142; danger of it, 143; its analogy to the small-pox, ibid.

CLAY SOILS, favourable to intermittents, i. 258.

CLAYTON, Mr. his case of ulceration of the larynx, xxii. 25.

———, Thomas, on the mischief arising from Ching's worm lozenges, xvii.

CLAYTONIA ALSINOIDES, a plant introduced into England from Nootka Sound, described, xxiv. 529.

CLEANLINESS, advantages of, ii. 394; consequences of the want of, iv. 12; instructions for attending to it in hospitals, xi. 97; necessity of enforcing it among the poor, xvi. 572; recommended to plumbers, glaziers, and painters, xxi. 526.

CLEGHORN, Dr. on epidemic diseases, xxi. 450; his practice of bleeding in fevers, xxii 459.

CLEMENT, Julian, his eminence as an accoucheur, xxviii. 102.

———, W. on herpetic affections, xiii. 415; reply to, xv. 307; xvi. 65; on the vaccine vesicle, 69.

CLEPHANE, Dr. his account of a preparation for the gout, v. 416.

CLERGY, advice to them on the study of medical botany, xxi. 101.

CLEVELAND, Parker, on the medical effects of electricity, xxxi. 37.

CLIFT, Mr. his account of a fractured spine, xxvi. 373.

CLIFTON, Gloucestershire, account of the influenza at, x. 224.

CLIMACTERIC DISEASE, observations on the, xxxi. 163.

CLIMATES, on the diseases of warm, viii. 276; on the intermittents of temperate, 277; directions on the change of, ix. 33; observations on the fevers of hot, x. 36; precautions to be adopted by Europeans in warm, 83, 280; on the diseases of hot, xi. 158, 200; xiii. 481; proper ones for consumptive persons, xi. 284; changes in that of England, 491; on the scurvy in hot, xiii. 481; on peculiar diseases incident to, ibid.; danger of incautious exposure to cold air in, 524; influence of cold ones, xx. 5; on diseases arising from changes of the, 7; effects on form and colour, 240; on the disorders incidental to Europeans in hot, xxi. 71; on the seasons of sickness in the same, 72; observations on the coast of Guinea, 73; on the unwholesome seasons and diseases of the East Indies, 74; rainy season in Bengal, 75; unwholesome seasons in the West Indies, 76; on the necessity of quitting impure air, 77; on regulating the temperature of, xxiii. 262; on that of Malta, xxv. 64; on that of Madeira, 425; its effects on the human frame, xxvi. 10; on the influence of, xxviii. 488; medical effects of, xxx. 129; on the utility of changing it for consumptive patients, xxxiii. 242; on that of the East Indies, 244; on that of Sicily, 355; on that of Egypt, 395; an artificial, xxxiv. 371.

CLINCH, Rev. Mr. his practice of vaccination, v. 401.

CLINE, Mr. sen. on cells in cancerous tumours, vii. 87; virtue of aloes as a vermifuge, 257; his evidence on vaccination, 496; on tying the arteries, viii. 3; case of vaccine inoculation, 17; his method of dividing the crural arch, xi. 120; his remedy for spasmodic strictures, xv. 156; improvement in the catheter, suggested by him, xvii. 463; his treatment of contused urethra, xxi. 368.

CLINE, Henry, jun. his method of securing ligatures upon arteries, xvii. 192; on improving the breed of animals, xviii. 7.

CLINICAL TABLES, forms of, i. 11; on the advantages of, 176; Institute at Wurtzburg, annals of the, 484.

CLITORIS, first described by Fallopius, iii. 157; case of an extaordinary enlargement of the, v. 1; that case common in Egypt, 3; method of extracting it, 4; case of morbid enlargement of the, xxv. 22; case of diseased, xxxii. 427.

CLIVERS, the efficacy of it in cancer, xxxii. 255.

CLOCK, substitute for one in the night, xv. 94; description of one constructed without weights, xviii. 579.

CLOSE-STOOLS, improvement in, x. 176; directions respecting, xi. 99.

CLOSSIUS, M. on the effects of decapitation, i. 51.

CLOTHIERS, a new oil recommended to, xx. 385.

CLOTHING, danger attending a hasty change of, iii. 504; on the neglect of clean, iv. 12; attention to it necessary in warm climates, x. 84; importance of attending to it in pulmonary diseases, xxxi. 371.

CLOTHS, excellent vegetable dyes for, xvi. 75, 170.

CLOUDS, on the formation of the, xvii. 485; their precipitation in rain, snow, and hail, occasioned by electricity, ibid.; remarkable instance of the bursting of one, xx. 384; causes of the elevation of the, xxv. 53; chemical combinations in, 149.

CLOUGH, Dr. his account of an extraordinary case of twins, xxv. 29; his proposed converzatione for medical students, 368; his case of embryulcia, 387; on a case of exomphalocele, xxvi. 40.

CLOVER, description of the bird's foot, xix. 163.

CLUSIUS, his account of the smoaking of tobacco, xxiv. 449.

CLUTTERBUCK, Henry, on the opinions of John Hunter, ii. 86; on an ambiguous case of hydrocephalus, 154, 247; case of tetanus, iv. 287; his case of erysipelas followed by anasarca, vi. 131; on the internal use of acetate of lead, viii. 209; defence of Mr. Hunter's ideas and language in letters to, 412; his inquiry into the seat and nature of fever, xviii. 74; on the opinions of, xix. 170; xx. 35; ascribes all fever to diseased brain, xxviii. 505; examination of his opinions, xxx. 180.

CLUYTIA ALATERNOIDES, description of that plant, xxiv. 530.

CLYSTERS, recommended by Hippocrates for colics and fevers, xii. 470; obstruction in the œsophagus removed by the tobacco, xvii. 30.

COAL, alleged to be a compound substance, iv. 185; experiments on the nature of, 469; quantity of carbon in, v. 468; death produced by burning peat, vii. 512; on the non-conducting power of, ix. 238; extraordinary disease among the labourers in a mine of, xv. 93; xvii. 472; method of conveying air into mines, xvii. 198; rocks between beds of lime-stone and those of, xxviii. 151; remarkable bed of, in Lancashire, 158; another in Yorkshire, 159; state of the pits in the north, ibid.; oil extracted from the Bovey, 235; on the management of horses used in that trade, xxxiv. 420.

COALBROOK DALE, remarkable appearances in the nodules of iron stone at, xix. 542.

COBALT, existence of the acid of, x. 94; method for obtaining pure metallic, 95; description of a metal connected with, xv. 390.

COBRA DE CAPELLO, analogy between its bite and that of a mad dog, xiii. 158.

COBWEB, its virtues ascertained in intermittents, xxi. 353; case of its efficacy, 355; on its extraordinary powers in allaying irritations, 357; on the medicinal uses of it, xxii. 95; cases of intermittents, in which it proved ineffectual, 218; defence of the use of it, with additional proofs, 369; cases of its success, 370, 374; on the early use of it in agues, 457; instance of its inefficacy in ague, xxiii. 212.

COCCINELLA SEPTEM PUNCTATA, a certain remedy in tooth-ache, iv. 82; tincture of it recommended, v. 547; myriads of that insect in Kent, xxi. 9.

COCHINEAL, an insect of that genus found in the hieracium, xix. 257; reward offered by the government at

Madras for the importation of the real, xx. 480; method of preserving it, ibid.
COCHLEA, its admirable uses in the production and conveyance of sound, xix. 413.
COCHRANE, Hon. Basil, his improvement in the construction of vapour baths, xxi. 85; observations on his invention, xxiv. 34, 360.
————, Hon. Capt. his method of making yeast, xxii. 406.
COCKS, observations on the training of game, xiii. 537.
COFFEE, prohibition of it in Sweden, iii. 270; substitutes for it in consequence, 271; chemical analysis of, v. 371; pestilence ascribed to putrid, vii. 311; analysis of, xx. 384; the beet-root substituted for it in France, xxii. 525; the yellow water-flag a substitute for, xxxii. 346.
COFFIN, Dr. his two cases of ruptured uterus, xxxiii. 359.
COGAN, Dr. on vaccine inoculation, vii. 251; his exertions in forming the Humane Society, xxiii. 337.
COHESION, on the want of, i. 157.
COKE, experiments on the conducting powers of, ix. 239.
COLBATCH, Dr. his observations on the use of the misletoe, xii. 33.
COLCHESTER, resolutions of the Medical Society there in favour of vaccine inoculation, vii. 528; xx. 565.
COLCHICUM AUTUMNALE, or meadow saffron, botanical description of the, xxiii. 426; preparation of the syrup of, 427; its alleged efficacy in gout, xxxii. 78; proper mode of taking it, 79; testimonies of its virtue, 164; objections made to it, 198; vindication of its use in gout, 201; its remarkable effects on cattle, 216; cases of its benefit in gout, 312, 393; further observations on its use, 430; on the mode of its operation, with cases, 491; beneficial effects of the tincture of it in gout, xxxiii. 264.
COLD, (considered as a temperature,) method of producing artificial, i. 196; petechiæ produced by, 421; mortification produced by immersing the hand in, v. 534; severity of it in London in 1802, vii. 556; attended with unusual symptoms at Paris, ix. 287; report on the same, 419; remarkable instance of its influence in inebrity, xi. 570; how the humours are affected by, xxiv. 69; method of producing artificial, 347; on the sensation of, xxv. 470; degrees of it borne by reptiles and insects, xxvii. 485; its extreme severity at Hudson's Bay, xxix. 404; a predisposing cause of mortification, xxx. 20; its effect in producing scrofula, xxxii. 499; peculiar effects of, xxxiii. 246.
COLD, (the complaint of a) fatal consequences of neglecting it, v. 16; effects of catching what is called a, vi. 94; quotation from Chandler's Treatise on the, xiv. 371; observations on the effects of catching a, xv. 91; on the impropriety of the term as applied to a disorder, xxvii. 498.
————, (medical use of) benefit of the effusion in fever, i. 18; iii. 51; in inflammatory cases, ii. 286; in scarlatina cynanchia, iii. 556; efficacious in fevers, iv. 25, 105; in amaurosis and weakness of sight, 179; in tetanus, 287; history of the application of it in medicine, 397; beneficial in typhus, 402; successfully used in fever, v. 13, 75, 194; efficacious in burns, 238; beneficial in dysentery, 490; on its use in old ulcers, vi. 19; in various diseases, 107; in angina inflammatoria, 108; in retention of urine, 109; in bilious cases, 110; communications on its efficacy by affusion, 331; observations on its use in typhus, viii. 158, 356; in ulcers, 206; in burns and scalds, 443; its alleged benefit in gout, vii. 454; ix. 116, 205; in typhus, 206; observations on the medicinal application of it, 210, 282; its use in gout, 362, 442; salutary in convulsions, 508; in cases in which the cold application was beneficial, ix. 547; another of its efficacy in rheumatism, 549; sudden relief in gout from, x. 255; in rheumatism, 256; in yellow fever, 271; observations on its utility in local inflammations, 377; in convulsions, 410; in fevers at sea, xi. 22; in scarlatina, 27; its inefficacy and danger in gout, 150; its use vindicated, 241; success of it in gout and rheumatism, proved by cases, 346; an extraordinary case of the benefit of it at sea, 523; objections to the refrigerating plan, xii. 64; defended, 216; proper in ophthalmia, 234; fatal in gout, 377; caution necessary to be used in its application, 502; successfully applied in convulsions, 508; beneficial in colic, xiii. 18; confirmation of its utility in gout, 142; on a case in which it proved fatal, 144; another, 229; observations on the case, 231, 355; on the casualties occasioned by, 437, 460, 512; a successful case, xiv. 120; observations on, 215; errors respecting it confessed, 456, 497; recommended in fever, xiii. 564; history of that practice, 566; rationale of the practice, xiv. 85, 179; its use in the influenza, 226; its effects on the human body, 239; salutary cautions on the use of it, 260; in trismus, 267; its efficacy in convulsions, xv. 52; danger of it in gout, 181; beneficial in burns and scalds, 342; its ill effects in such cases, 458; successful in tetanus, 497; objected to in scalds and burns, xvi. 8; defended, 17; farther objections to, 115; vindicated, 210; remarkable cases of its success in typhus, 236; a sedative remedy, 366; difficulty of ascertaining where it actually commences, ibid.; general effects of a diminished temperature, 367; its effects on the pulse, ibid.; on violent degrees of it, ibid.; on the application of it to the human body, 368; opinion of Darwin on that subject, ibid.; history of its medical application, 370; cautions on its use, ibid.; its benefit in various diseases, 371; case of obstinate headache cured by, 372; serviceable in cancer, 383; additional cases of its efficacy in burns and scalds, 461, 465, 526; in yellow fever, 490; moderation recommended in its use, xvii. 1; observations on the practice, 21; beneficial in hernia, 55; its effects on the muscular fibre, 93; on the refrigerant plan in burns, 133; remarks on the efficacy of that treatment, 320; bad effects of it in gout, xviii. 52; humorous account of the practice in scalds, 67; observations on the same, 160, 163; on its use in fevers, 197; its virtue proved in burns and scalds, 208; and in gout, 240; the cause of tetanus, 261; remarks on the utility of it in burns, 360; recommended in hydrophobia, 400; topical refrigeration in scalds objected to, xix. 17; its use in rousing excitability, 112; on its use in fever, 175; advantage of it in small-pox, 272; its effect in rousing excitability, 304; in scarlatina, 320; its impropriety in gout, 572; remarkable instance of its success in fever, xx. 51; defence of its use in gout, 65; exposition of its use in fever, 81, 164, 166, 169; considered as the cause of rheumatism, 357; obligations to Dr. Currie for its medicinal application, 562; objections to it in burns, xxi. 387; a preventive of diseases in hot climates, xxii. 230; salutary effects of topical, 233; its efficacy in nasal hæmorrhage, 327; cases in which it failed to be of service in fever, 431; its effects in allaying thirst, xxv. 473; rationale of its operation in gout, xxvi. 96; proved beneficial, 97, 369; directions for its application externally, 397, 431; its indiscriminate use deprecated, 433; benefit in scarlatina, 445; emphysema cured by the application of, xxviii. 141; in peritoneal inflammation, xxx. 19; beneficial in inflammation of the viscera, xxxii. 90; efficacious in typhus and scarlet fever, xxxiii. 358; on the treatment of gout by, xxxiv. 6, 184; vindication of that practice, 361.

COLDHAM, Mr. his reports of the vaccine institution at Nottingham, xvi. 136; xxiv. 524.

COLE, Dr. John, on the frequency of apoplexy, xxviii. 218.

———, T. C. his account of a case of tetanus successfully treated with opium, vii. 55.

——— RAPE, experiments to extract sugar from, iv. 348.

——— WORT, description and virtues of the sea, xviii. 173.

COLEMAN, Edward, his singular case of extra-uterine conception, ii. 262; his observations on the contagious glanders in horses, xxiii. 215; his method of treating pulmonic complaints in horses, xxv. 281; his observations on the lungs of broken winded horses, 341.

COLEY, James Millman, his case of an enlarged and tuberculated liver, xviii. 485; case of scirrhous ovary, with an adipose tumour containing hair, xxiii. 237; on diseases of infants, xxx. 237; his case of urinous vomiting, 465.

———, William, his case of tympanites in an infant, vii. 223; remarks on the case, 228; his critical analysis of opinions of different writers on the subject of tympanites, 230; on acupuncture, 236; his case of anasarca and hydrops pulmonum, 406; account of the ascites and hydrops vaginæ, 412; case of inoculated cow-pox succeeded by confluent eruption, xi. 385.

COLIC, instance of its being occasioned by the use of lead spoons, iii. 90, 167; violent at Madrid, 167; benefit of oil in, vi. 70; varieties in that disease, vii. 508; efficacy of alkalis in, viii. 293; cases where the saturnine colic originated in solutions of lead, xi. 571; benefit of the cold affusion in, xiii. 18; its frequency in the Highlands of Scotland, 36; efficacy of linseed in, xiv. 63; the flatulent cured by opiates, 271; benefit of peppermint in, xvii. 560; seeds of the tansy efficacious in it, xix. 354; observations on the saturnine, xx. 342.

COLICA PICTONUM (Devonshire Colic), said to be occasioned by the use of lead, vii. 508; observations on that complaint, xi. 463; case of the success of nicotiana in, xii. 403; benefits of calomel in, xxvi. 44; on the use of nitric acid in, 45.

COLLA, Professor, his cases of the success of cannabina in tænia, xxiv. 517.

COLLECTANEA MEDICA, (containing anecdotes, extracts, &c. illustrative of medical science,)—veracity in reporting facts, xxvii. 115; success of Sir Hans Sloane in treating the small-pox, ibid.; state of physicians in 1670, ibid.; state of surgery in Scotland in 1585, 116; account of Andrew Borde, ibid.; partnership between physicians and apothecaries, ibid.; on the classical knowledge of Sydenham, 117; anecdote of Dr. William Hunter, ibid.; early practitioners of midwifery, ibid.; on the term man-midwife, 118; account of Dr. Bruyn's secret, ibid.; inoculation for the measles, ibid.; permanency of morbid contagion, ibid.; diseases of Iceland, ibid.; diet of the Icelanders, ibid.; leprosy in that country, 119; singular complaint there, 123; experiments on cantharides, 126; experiments on camphoric acid, 127; observations on the Zetland sheep, 216; on the composition of writing inks, 224; extraordinary instance of trance, 227; essay upon cinchonin, 295; historical account of the foundation of the royal hospitals, 306; on the production and ripening of the seeds of plants, 310; attachment of an idiot to bees, ibid.; tobacco and snuff manufacture of New Spain, 311; anecdote of Mrs. Kennion, midwife, 380; anecdote of Dr. Frank Nichols, 381; on the muscular coat of the bladder, ibid.; superstition among lying-in women, ibid.; guttural tumours in Hindostan, 382; strange error of Fourcroy, ibid.; account of the mermaid of Haerlem, ibid.; reception of vaccination by the North American Indians, 383; account of the German universities, 385; on certain vegetable muscicapæ, 388; degree of cold suffered by reptiles and insects, 485; account of the jumping ague in Scotland, 486; of bleeding in the side affected, 488; on the strength of men and horses in moving machines, ibid.; memoirs of Dr. Maxwell Garthshore, xxviii. 42; privileges of the College of Physicians, 49; extracts from the College annals, 52; account of the elastic gum or caoutchouc, ibid.; action of poisons on the animal system, 115; licence of practice from Archbishop Sheldon to William Lilly the astrologer, 129; preparation of soporific medicines from garden lettuce, 130; on vegetable wax, 136; history of the art of treating the deaf and dumb, 193; comparative statements of the healthiness of London, 195; compound of carbonic oxide and chlorine, ibid.; appearance of a volcano off the western islands, 200; analysis of deadly nightshade, 205; observations on fossils, 304; process of petrifaction, 305; population of Great Britain, 307; regulations for pupils at St. George's Hospital, 311; chemical researches on the blood, 386; analysis of the urine of the ostrich, 470; method of obtaining artificial blood, ibid.; analysis of a rose-coloured substance found in urine, 471; of the lymph in the ventricles of the brain, 472; anti-syphilitic tincture of M. Mesnard, 473; account of another anti-syphilitic remedy, 475; account of aphrodisiacs, ibid.; Chinese knowledge of medicine, 476; efficacy of the eupatorium perfoliatum, ibid.; on blood-letting in typhus, ibid.; memoir of Dr. Robert Willan, 477; queries respecting India, 487; connexion between the formation of the head and the mind, xxix. 48; Suvorof's rules for diet, 49; influence of the brain on animal heat, 141; account of the psylli or serpent charmers, 304; foundation of the College of Physicians, 306; union of surgeons and barbers in one company, 307; punishments of quacks, 308; humiliating condition of apothecaries in the 16th century, 310; memoir of the Hon. Henry Cavendish, 397; account of the liquid gum of Botany-bay, 474; description of an organ in the eyes of birds, 481; of the cerebral matter of man and other animals, xxx. 55; on the medical effects of climates, 129; memoir on the action of vomiting, 207; state of vaccination in India, 216; cow-pox long known among the Bramins, 217; study of anatomy by the Rajah of Tanjore, 219; oriental practice of physic, 221; account of Goderneau's powder, 222; warm-baths at Paris, 223; on the heat evolved during inflammation, 323; effects of magnesia on urinary calculi, 324; proportions of alcohol in fermented liquors, 392; observations on radiant heat, 396; on the specific heat of different gases, 399; memoir of M. de Fourcroy, 499; extraordinary abstinence in the reign of Edward III. xxxi. 50; on the diseases of the Laplanders, 51; tables of the mean temperature at Stockholm and Petersburgh, during the same periods, 54; on the quantity of rain in Sweden, ibid.; state of vaccination at Norwich, 136; state of medicine in Spain, 235; practice of medicine among the Patagons, 304; practice of midwifery among the Indians of South America, 305; singular disorder in the crew commanded by Sir Thomas Cavendish, 306; case of diseased cervical vertebræ, 307; on the use of the epiglottis in deglutition, 318; on impeded deglutition, 392; case of suppression of urine from stricture,

398; memoir of M. Cabanis, 400; medical practice and diseases at Tripoli, 478; report of diseases in Christ's Hospital, 479; case of fractured cranium with laceration, 485; influence of the nerves on secretions of the stomach, 486; effects of oxygen gas in restoring suspended animation, xxxii. 36; case of a man who vomited a urinary kind of liquid, 41; case of retention of urine cured by puncturing the bladder, 43; proofs that the yellow fever is contagious, 45; use of venesection in diabetes mellitus, 128; on the influence of the temperature of the air in respiration, 132; fatal case of hydrothorax, 134; case of sudden death in child bed, 135; fatal case of hydrophobia, 217; case of tic doloureux cured by tar, 219; case of rheumatism of the heart, 220; account of the chemical discoveries of Berzelius, 222; extrication of caloric during the coagulation of the blood, 223; memoir on the eyes of insects, 314, 400, 500; case of tænia in an infant cured by the decoction of pomegranate, 395; observations on pemphigus, 397; on insects as the cause of the itch, 496; on certain combinations of chromic acid, with different bases, xxxiii. 33; answer to Mr. Hume on barytes, 35; experiments and observations on iodine, 37; memoirs of Dr. William Harvey, 108; experiments on animal heat, 113; on the combustion of the diamond and other carbonaceous substances, 121; on the Goitres of Savoy, 209; history of the Cagots, 211; account of a fall of uranolytes or ærolites, 215; answer to Mr. Phillips on barytes, 218; state of medicine in France during the year 1814, 298; account of Egypt, 391; description of the pyramids, 392; on the climate of Egypt, 395; proceedings of the French Institute, 396; cruelty of the French physiologists, 401; on the treatment of fungus cerebri, 403; official report of the French Institute, 471; on the plague at Malta, 477; improved method of making antimonium tartar, 484; easy mode of procuring potassium, xxxiv. 26; apology for Mr. Hunter's supposed obscurity, 29; memoirs of Dr. Joseph Black, 36; on the economy of heat in distillation, 114; case of popliteal aneurism cured by an obliteration of the femoral artery, 116; case of axillary aneurism, in which the subclavian artery was tied, 118; etymology of the small-pox, 121; uncertainty of its early history, 125; description of improved crutches, 128; memoir of Smithson Tennant, esq. F.R.S. 202; instance of small-pox infection from a subject that had been buried thirty years, 225; account of the epidemic at Gibraltar, 301; Dr. Spurzheim's remarks in answer to Dr. Baillie and Sir Everard Home, on the structure of the brain, 309; prize essay on the hospital gangrene, 394, 467; account of the pestilential disorders in Andalusia, 406; observations on the sirocco, 408; pestilential winds of Africa, 409; on the influence of those winds in producing epidemics, ibid.; sketches of the medical schools and hospitals at Paris, 478; description of L'Hotel Dieu, 479; account of the Hospice de la Maternite, 487.

COLLES, Abraham, his observations on surgical anatomy, xxvi. 398; on tying the subclavian artery, xxxiii. 221, 378.

COLLIER, Charles, his case of poison by laudanum, v. 164.

———, Mr. his process for purifying fish oil, i. 86; on purifying water, 402.

COLLIERY, explosion in that of Collingwood Main, xxx. 427.

COLLINS, Dr. his remarkable case of tympanites, xiv. 267; on tinct. ferri muriati in retention of urine, xvi. 250; his case of diseased kidney, xvii. 189; case of supposed labour, in which a mass of hydatids was expelled, xxxiii. 346.

———, J. on the benefit of opium in labour pains, xxx. 303.

COLLINSON, Peter, memoir and anecdotes of, xxvi. 470.

COLLOMB, M. his medical and chirurgical essays, i. 104.

COLLOT, his operation for the stone, ii. 54; xi. 9.

COLLYRIA, forms of good ones for ocular inflammation, vii 215.

COLOCYNTHIS, used by way of friction in mania, xxvi. 317.

COLOGNE, account of the dispensatory of, xxii. 272.

COLOMBO ROOT, its virtues as a remedy for indigestion, xx. 193; part of the composition of the eau de medicinale of Husson, xxiv. 353.

COLON, Dr. on the inoculation for the cow-pox, v. 386, 556.

——— IN THE RECTUM, case of invagination in the, xi. 115; case of stricture of the, xxviii. 139.

COLORIFICATION, new theory of, xxviii. 335.

COLOUR, observations on the varieties of, in mankind, i. 406; in man not changed by climate, xx. 240; on the peculiarities of it in the human species, xxii. 8; extraordinary case of the change of, xxv. 24; on that of the blood, xxviii. 397; on the theory of, 335.

COLOURS, how to preserve those of flowers in a dry state, iii. 91; causes of the phenomena of, 501; experiments on several specimens of, found at Pompeii, xxiii. 84; on changes effected in, 350; considered as re agents, 351; of plants, observations on, xxvii. 18.

COLTHORPE, Lincolnshire, recipe for the bite of a mad dog found in the church at, xiii. 297; observations on that recipe, xiv. 33.

COLTS' FOOT (*Tussilago*), recommended in consumption, ii. 71; its medicinal virtues, xix. 359; when smoked good for coughs, 360; good tinder made from the leaves, ibid.

COLUBER CARCINATUS, description of the, xxviii. 155; effects of its bite, 156.

COLUBER NAGA, remarkable effects of the bite of the, xvii. 295; case of a person recovered after being bitten by one, xxvii. 432.

COLUBER NATRI, a snake in Italy, extremely fond of milk, xiii. 287.

COLUMBIA COLLEGE, New York, lectures at, i. 88.

COLUMBIUM, discovery of a new metal so called, vii. 477.

COLUMBUS, Realdus, anatomical professor at Padua, his character, ii. 66; account of his works, ibid.; claims the discovery of the stapes, 160; traces the connexion of the nerves to the muscular fibre, 163; on the omo-hyoideus, 165; his error respecting the vena cava, 167; pretends to have discovered the circulation of the blood, 368; his defence of Galen, 460; his description of the duplicatures of the peritoneum, iii. 73; of the pancreas, 74; his error respecting the pleura, 77; description of the ventriculus laryngis, 78; censured for obscenity, 157; described the hymen, 158; on the semen in females, 159; his opinion on the third pair of nerves, 358; on the massetericus, 361; his description of the first cervical nerve, iv. 342.

————, Christopher, his discovery of gold in Hispaniola, xxv. 509.

COLYMBUS IMBER, or the ember goose, account of the, xxvi. 168.

COMA, gout productive of, ii. 377; observed in epidemics, ix. 386; observations on, xxviii. 211.

COMBE, Dr. Charles, his case of stricture and thickening of the ileum, xxxi. 71.

COMBINATIONS, observations on galvanic, xxi. 350; on that of phosphorus and sulphur, xxviii. 157; farther experiments on the same, xxx. 83; instance of combustion during that process, 523; on some unknown ones, xxxiii. 33; on detecting iodine in, 47.

COMBUSTION, takes place only in oxygenous gas, i. 168; phenomena attending that of organized bodies, 169; calcination of metals produced by, 170; the theory of it not known till the 17th century, 253; on that of phosphorus, ii. 389; on that of the human body, from spirituous liquors, iv. 44; why peculiar to women, 45; the clothing not injured in such cases, 46; farther observations on that of the human body, xii. 87; remarkable instances of it, 88; on that of charcoal, xxiv. 347; spontaneous in manufactories, xxviii. 514; that of a woman, xxx. 164; instance of it in the combination of phosphorus and sulphur, xxx. 523; on that of the diamond, xxxiii. 121; spontaneous in a defunct drunkard, 425.

COMET, longitude of that which appeared in 1770, xxv. 362; account of that of 1811, xxvi. 351; observations and description of the same, 429; on the nature of, 430; luminous body round the nucleus of the, xxvii. 168; discovery of a new one, ibid.; Dr. Herschel's observations on the, 256.

COMFREY, recommended in consumptions, ii. 71; botanical description of, xii. 125; good in diarrhœa, 126.

COMMIPHORA MADAGASCARENSIS, description of that tree, and the caoutchouc produced by it, xi. 399.

COMPRESSES recommended in tumefied intestines and hernia, ii. 46.

COMPRESSION in apoplexy, on, vii. 207; on that of the arteries, x. 156; Camper's experiments for, 157; heat disengaged by the air in that of a wind gun, xii. 575; case of that of the brain, xxxii. 8; its utility in rheumatism, xxxiv. 196.

COMPRESSOR ARTERIÆ, description of an improved, xxxiv. 3.

COMSTOCK, Dr. his case of singular nervous affection, xx. 224.

CONCEPTION, case of extra uterine, ii. 481; singular opinion of the ancients on, iii. 160; case of extra uterine, vi. 360; remarkable cases of, xvii. 492; observations on mental, xxi. 496; on the progressive state of, xxiii. 280; instance where the hymen was unbroken in, xxiv. 232.

CONCRETIONS, analysis of arthritic ones, i. 194; analysis of urinary, 448; analysis of a remarkable gouty one, iii. 174; case of calculous ones in the tonsils, 446; stony ones coughed up from the lungs, x. 327; on bony ones out of the usual course of nature, xiii.

505; on certain animal, xvi. 3; on urinary, ibid.; biliary, ibid.; on intestinal ones, 4; on the nature of gravelly and calculous, 153, 221, 305, 497; calculous one of the eye, xvii. 83; on gouty ones, xxii. 155; on atmospherical, 179; efficacy of carbonate of potash in urinary, xxiii. 45; found in the lungs of consumptive persons, xxv. 337; obstructions in the alimentary canal occasioned by alvine, xxvii. 501; account of one found in the cavity of the thorax, xxx. 73; large collection of urinary, xxxi. 81; case of olive oil and mucus found in that state, 45; one found in the jejunum, xxxii. 80; dumbness occasioned by, 169; xxxiii. 172.

CONCUSSION, case of that of the brain, with observations, ix. 272; other cases of the same, xiv. 486, 490; its effects on the mind, xxvi. 131; case of, xxix. 460.

CONDOR, of South America, on the enormous size of the, xxvi. 15; supposed to have been the gryphon of the ancients, ibid.

CONFORMATIONS, on vitiated ones of the human body, xxi. 238, 282.

CONGESTIONS, observations on various, xxi. 226; in the lungs and head, 300.

CONIUM MACULATUM, its effects in venereal complaints, xiv. 125; its roots mistaken for parsnip, xx. 15; beneficial in cancer, xxi. 11. See *Hemlock*.

CONNOR, Dr. his notice of Mayow, i. 221.

CONQUEST, Mr. his case of malformation of the genital organs, xxv. 441.

CONRADI, Dr. on two species of the cramp in the stomach, i. 49; his treatment of cataract, xxx. 71.

CONSBRUCH, Dr. on the use of snails in fistulous ulcers, ii. 75; his account of hereditary hæmorrhages, xxvi. 422.

CONSCRIPTS, regulations for passing them to the French army, xxiii. 429.

CONSERVE, how to prepare that of wood-sorrel, vii. 569; xv. 267; of roses, how made, xvi. 262; benefit of that of spearmint, xvii. 539.

CONSTANT, Mr. his method of refining sugars, xxix. 249.

CONSTANTINOPLE, state of vaccination at, v. 243; viii. 95; on the epidemic pestilential fever at, ix. 502; vaccine inoculation found a security against the plague at, x. 474; observations on the plague at, xv. 113; number of medical practitioners at, xxvii. 274.

CONSTIPATION, case in which it was removed by external friction, xv. 325; cured by olive oil, xix. 51; obstinate case of, xxi. 480; attended with delirium, 484; produced by swallowing cotton, xxiii. 431; case of the cure of, 500; case of, xxx. 441; advantage of opium in, 441.

CONSULTATIONS, observations on the neglect of medical, xix. 204; necessity of them, 205; advantageous to the profession, 206.

CONSUMPTION, on the supposed benefits of digitalis in the cure of pulmonary, i. 290, 291, 383; ii. 35, 102, 113, 175; frequency of it in London, i. 336; efficacy of green broom in, 383; on the causes and prevention of, 410; notes subjoined to a translation of Dr. Reid's Essay on, ii. 50; historical facts relative to the cure of, 70; effects of digitalis in, 238; cases of tubercular, 267; objections to the digitalis in, 314, 319, 430; on the benefit of living with cows for persons in, 415; on the cure of, 417; of the medicinal power of digitalis in, iii. 121, 127, 128, 202; fancied to be contagious, 274; case of the effect of digitalis in, 311; on the management of, 312; cases continued, 313, 315; Dr. Carson on the advantage of digitalis in, 513; considerations on pulmonary, 560; on the use of digitalis in, iv. 127; advantage of the warm bath in, 148; Dr. Mossman's observations on, 308; cases of, 311; Dr. Drake on the efficacy of digitalis in, 521; semina phillandrii aquatici, said to be good in, v. 95; observations on the effects of digitalis purpurea in, 201; cases of, 204, 213, 260; observations on pulmonary, 307; considerations regarding pulmonary, vi. 89; cautions respecting visiting Madeira, in patients afflicted with, 415; success of digitalis in, viii. 82; frequent in Lincolnshire, 224; observations on the causes of, 225; yet rare in Holland, 226; case of, 227; successfully treated by salivation, 308; observations on pulmonary, 463; the lichen islandicus recommended in, 465; on the use of that remedy in, ix. 19; remarkable case of, owing to mastuabation, 56; benefit of opium in, 270; benefits of the Lichen Islandicus proved in, x. 379, 380; case of the success of digitalis in, 551; others in which it was unavailing, 554; affecting picture of, 572; use of digitalis vindicated in, xi. 59; observations on a case of, 147; on the Lichen Islandicus as an aliment and remedy for, 283; Devon and Cornwall recommended for patients in, 284; digitalis successful in three cases of, 286; correction of an error respecting the use of lichen islandicus in, xii. 463; case in which the digitalis proved a cure, xiii. 323; cases of the success of uva ursi in, xiv. 463; on the tubercles in the lungs in, xv. 30; method of cure in, 32; cases in which the uva ursi effected a cure, 346; on the origin, progress, and treatment of, 460; cases of the efficacy of digitalis in, xvi. 231, 233, 353; strawberries said to be good in, 264; frequent in America, xvii. 79; prize question relative to, 199; on the use of the Iceland liverwort in, 255; horehound a remedy for, 564; its prevalence in England, xviii. 9; on the uva ursi in, 10; Dr. Reid's inquiry into, 11; its frequency at Paris, 289; cases in which mercury was effectual, 324; effects of labour in the cure of pulmonary, 448; cured by mercury, xix. 20, 219, 224, 227; use of opium, cordial drinks and animal food in, 47; account of Dr. Saunders's work on, 263; on the use of digitalis in, 268; on the use of emetics in pulmonary, 337; deaths in London by, 338; fat an occasional cause of, 339; cases of, 340, 343, 344; elecampane serviceable in, 455; salutary effects of salivation in pulmonary, 506, 508, 510; benefit of digitalis in, 511; practical remarks on the use of that remedy, 515; on the action of digitalis in, xx. 13; on the virtue of Lichen Islandicus in, 268; case of, 409; observations on dissections, 411; on the efficacy of rest in, xxi. 327; xxii. 89; frequent in Devonshire, 106; organic injury of the intestines producing symptoms of pulmonary, 150; account of the institution at Bristol for the cure of, xxi. 188; on the infectious nature of, 193; illustrated by a case, 197; an hereditary disease, 283, 292; on the use of digitalis in, 328; its ravages at Nottingham, 425; proportional mortality from it in different parts of the kingdom, xxii. 239; on two species of, 455; necessity of a regulated temperature in the cure of, xxiii. 150, 262; on the opaque matter expectorated in pulmonary, 221; on the connexion between it and amenorrhœa, 238; on a regulated temperature for the cure of, xxiv. 21; concretions found in the lungs of a person who died of, 337; cases of the efficacy of regulated temperature in pulmonary, xxvii. 159; xxix. 376, 391; remarks on those cases, xxx. 49; cured by carbonate of barytes, 466; importance of attending to clothing in, xxxi. 371; on the nature and treatment of, xxxiii. 207; regulated temperature vindicated in, 315, 365; letters to the Duke of Kent on, xxxiv. 68; Dr. Southey's Treatise on, 233; prevalent in Sicily, 359; Dr. Buxton's reply to Dr. Sutton on, 368.

CONTAGION, on its extirpation, i. 80; on the nature of, 201, 202; effect of nitrous vapour in destroying, 206; conveyed from St. Domingo to Philadelphia, 207; little understood, 355; on the means of destroying, 408; observations on, 470; on fumigation in destroying, iii. 245, 321; vindication of that practice against Dr. Trotter, 429; testimony in favour of that plan, 484; Dr. Trotter in reply, 526; on the matter of, iv. 114; generated in the houses of the poor, 395; on variolous, 518; v. 81, 146; causes of the prevalence of, 97; on the cure and prevention of, 185; in the metropolis, causes of the, 289; on the means of self-preservation in, 582; on preventing it among the poor, 589; benefit of oil against, vi. 71; on the prevalence of typhoid, 198; warm weather favourable to, 365; on that of marshy situations, 493; observations on febrile, 499; gunpowder recommended to destroy, vii. 39; on that of marshes, 113; on that of fever, 255; prize question on, 300; historical view of cases of, 311; state of the atmosphere in, 317; washing with vinegar recommended in, 341; engendered in crowded ships, 381, 383; on the discovery of the power of nitrous fumigation in checking, 424; on the means of preventing, viii. 185; on the discovery of muriatic fumigation as a remedy against, 187; calcareous regions free from, 223; on the means of purifying the atmosphere from, 529; plan for preventing it in London, 538; on that of the yellow fever, ix. 29, 103; on the precautions necessary as a security against, 182; on establishing pest-houses to diminish, 504; catarrhal fever supposed to partake of, 527; on the discovery of the power of acid vapour in destroying, x. 78; communications to decide the question of, 97, 126, 127, 129, 193, 231, 232, 233, 289, 312, 385; on that of the scarlet fever, 458; restrictions requisite to prevent, 460; that of the influenza questioned, xi. 81; the action of it local, 82; instructions for preventing it, with an apparatus, 97; mechanical means, 100; chemical means, 103; on the effect of nitrous vapours in preventing, 136; virtues of muriatic acid in destroying, 292; propagated by air, diseased persons, and goods, 491; the Egyptian ophthalmia communicated by, xii. 79; denied with regard to the plague, 92; effects of nitrous acid gas in, 484; on the causes of, xiii. 110; means of curing, 112; proposals for preventing, 114; on preventing the return of, 116; proceedings of the society for preventing, 190; obscurity respecting the origin of, 210; outlines of a plan for putting a stop to, 272; report of the London Institution for preventing, xiv. 340; prize questions on the nature and prevention of, 381; instinct of sparrows in discovering, 382; inquiry into that of the yellow fever, 385; Gaubius on the means of preventing, 573; recantation of Dr. Rush on, xv. 271; examination of Morveau's claim to the discovery of the power of acids against, 279; Dr. Johnstone's claim to that discovery, 373; new theory on, 571; experiments made in Spain on acid fumigations against, xvi. 190; its cessation in London, 288; observations on, xvii. 101; on diseases which really have that character, 103; denied in regard to yellow fever, 105, 112, 115, 120; Dr. Chisholm on, 128; Dr. Alder in refutation of the doctrine of, 505; denied with respect to the plague, 506; on that of Egypt, xviii. 13; difference of opinion in America respecting, 42; on the true nature of, 88; instances in England of, 89; observations on, 112, 424; on specific, 472; that of fever denied, 497; the doctrine of exploded in America, xix. 113; general view of its causes, 199; review of the controversy on, xx. 27; observations on the agency of, 30; on the use of the mineral acid gases in, 35; on epidemic, 366; facts to establish the certainty of, xxi. 77, 241; difficulties that embarrass the question, 91, 299; dissections of persons who died of, 453, 456; on the nature of, xxii. 36; confounded with epidemical diseases, 105; on that of the sore throat, 176; divided into different classes, 513; efficacy of tobacco in checking, xxiii. 118; on the various kinds and degrees of, 177; prize question of the Medical Society at Paris, on the nature of, 260; on diseases that spread by, 473; on the animalcular theory of, xxiv. 23, 25; that system supported by Linnæus, 26; efficacy of tobacco against, 136; effects of dead bodies in producing, 422; on that of inoculated small pox, xxvi. 117, 207; permanency of morbid, xxvii. 118, 142; croup and scarlatina maligna, communicated by, 199; on the effects of oxygenated muriatic acid against, 408.

CONTAGIOUS DISEASES, on the re-appearance of, x. 177; origin of, 461; the influenza so considered, xi. 81; observations on, xiii. 110; yellow fever denied to be of that character, 378; herpes not contagious, xvi. 179; the Egyptian ophthalmia considered as such, xviii. 12; the yellow fever denied to be of that description, 42; observations on, 92, 111, 424; on the proximate causes of, xix. 198, 334; elecampane said to be serviceable in, 455; general view of, xx. 27; observations on their character, xxi. 77, 454; on the causes of, xxii. 36; on the treatment of, 459; memoir on a prevalent one in France, xxiii. 33; treated with stimulants, 37; account of a remarkable one among cattle, 167; general remarks on, 168, 170; the hooping-cough considered as such, 178, 180; chronological retrospect of, 282, 288; account of a remarkable one, xxiv. 365; report on one at Edinburgh, xxvii. 166; the croup and scarlatina considered under that denomination, 200.

——————— MIASMATA, on the means of destroying, i. 429; question concerning, viii. 478; on the means of freeing the atmosphere of, 535; plan for the prevention of, 538; on the nature and causes of, x. 467; in America, cases of, xxiv. 365.

CONTE, M. his method of preventing the rusting of iron and steel, xi. 286.

CONTRACTIONS, experiments on galvanic, ix. 146, 235; x. 53; on those of the lower limbs, xv. 326; on muscular, xxi. 457.

CONTREXVILLE, analysis of the mineral waters of, vi. 186.

CONTROVERSY, on the benefits of temperate, vii. 260, 368; observations on, 371; on that relating to vaccination, xviii. 257, 318; advantages of it when properly conducted, 420; qualified explanation on, 537; on regulating it, xix. 331; necessity of, xx. 520; want of candour in the vaccination, xxi. 121; duty of moderation in managing, 331; intemperance condemned in, 333; account of the Liverpool one on a case of poison, 336, 426; remarks on that case, 399.

CONTUSION, on the general treatment of, xiii. 386; of the brain, cases of, xiv. 486, 487; observations on, 489; observations on, as a consequence of disease, xxiii. 369; case in which it was treated on the depleting system, 371; case of, attended with retention of urine, xxiv. 381; of the peritonium successfully treated, xxviii. 246.

CONVALESCENCE, danger of cutting the hair of patients in a state of, xii. 367; plan of an asylum for patients in a state of, v. 305; on the utility of bleeding in slow, xxviii. 132.

CONVALLARIA, botanical description and medicinal uses of the different species of, xiv. 66, 67.

CONVOLVULUS, or great bindweed, botanical description of the, xii. 129; its virtues as a cathartic, ibid.; *Convolvulus Soldanella* applied to swellings of the feet, ibid.

CONVULSIONS, case of puerperal, iii. 449; efficacy of alkali in, iv. 183, v. 558; remarkable case of, in childbirth, v. 473; of children, 475; cases of, 557; how excited in dentition, 571; on the cause of them in pregnant women, vi. 280; said to have been cured by the snake-root, viii. 428; on a case of, supposed to result from vaccination, ix. 532; efficacy of cold water in, x. 410; xii. 508; benefit of misletoe in, xii. 33; remarkable cases of, 112; singular cases of, 572; cases of the efficacy of cold water in, xv. 52; remarkable case of the effect of bleeding in, xvii. 549; those of wounded soldiers cured with opium and carbonate of potash, xviii. 94; chorea, a species of, 216; hydrophalus, not an effect, but the cause of, 219; cases of puerperal, 353; the puerperal occasioned by pressure of the brain, xx. 192; the same proved by cases, 493; occasioned by the bite of a rabid animal, xxi. 140; instance of hereditary, 237; the effects of laborious dentition, 286; on epileptic, xxiii. 18; considered as a consequence of disease, 367; case of, connected with great constitutional debility, 371; cases in which they were produced by diseases, 480; xxiv. 52, 57, 89; occasioned by the eau de medicinale, xxiv. 474; case of the occurrence of, during labour, xxx. 36; remarks on puerperal, 111; case of puerperal, 455; benefit of nitrate of silver in, xxxi. 148; remarkable history of the effects of fanaticism in producing, 373; explanation of that case, 564; xxxii. 32.

COOK, Edward, his observations on the effect of nitric acid in elephantiasis, xvii. 189.

COOK, Captain, his method of preserving the health of seamen in long voyages, i. 91; vi. 498; his observations on venereal disease, xvi. 173; on luminous appearances of the sea, observed in the voyages of, xxv. 409.

COOKE, C. on a spurious kind of cow-pox, i. 323; on a case of malformation, iii. 497; his recantation on the subject of the cow-pox, iv. 20; on the use of vinegar in contagious fever, vii. 255, 341; on the influenza, x. 197.

———, Rev. James, his method of preserving yeast, xxii. 404.

———, Mr. of Brentford, his case of hydrocephalus internus, xxviii. 58.

———, Robert, his case of incontinence of urine, x. 423; on the treatment of erysipelas, xii. 523.

———, William, his case of torpor, xxvii. 418; complicated case of cancerous stomach, xxx. 337; case of hœmatemesis, 513.

COOKSON, Dr. his case of premature puberty, xxiv. 117.

COOPER, Astley, his case of hydrocephalus, ii. 253; on perforating the membrana tympani as a cure of deafness, iv. 570; on the bursting of an absorbent, vi. 222; on the absorbents of the horse, vii. 139; on the contractile power of the absorbents, 221; his method of tying an artery in aneurism, viii. 1; his treatise on the treatment of inguinal and congenital hernia, xi. 279, 372; on a fungous tumour in the abdomen of a female, xiv. 79; observations on his treatment of hernia, xx. 3; his directions for reducing crural hernia by the taxis, 4; case of strangulated hernia, 261; his case of aneurism of the aorta, 480; on his treatment of crural hernia, 511; his dissection of a rabid dog, xxi. 273; his cases of aneurism of the carotid artery, xxii. 67, 69; on his practice of perforating the tympanum, 191; his operation on the spine of a dog, 164; experiments on the arteries of dogs, xxvi. 82; his case of popliteal aneurism, when the operation had been performed many years before, xxviii. 152; on the treatment of spina bifida, with cases, 153; his case of premature puberty, xxxii. 145; on the temperature of the heart, xxxiii. 117.

———, G. his observations on a case of pregnancy where the vagina was imperforate, xiv. 246.

———, Samuel, on the radical cure of bubonocele, ix. 161, 367; reply to, 396; on double vision, xix. 311; on diseases of the joints, xx. 4; his mode of treating crural hernia, ibid.; his case of hæmorrhage from the urethra, xxii 341.

COOTES, Henry, his case of epilepsy cured by trepanning, xvi. 473.

COPAL, easy method of obtaining the solution of, xii. 93; method of preparing varnish from, xiv. 382; a colourless varnish obtained from, xxii. 351; best menstruum for dissolving gum, xxviii. 432.

COPE, George, on a singular termination of the hæmorrhoids, xxii. 488.

COPLAND, Peter, on the lithontriptic power of the muriatic acid, ii. 34; xv. 570; xxv 446.

COPELAND, Thomas, on the principal diseases of the rectum and anus, xxiv. 415; vindication of, xxvii. 416; his case of calculus in a tumour of the groin, xxx. 170; his observations on the diseases of the rectum and anus, xxxiii. 236; on the symptoms and treatment of diseased spine, 406.

COPENHAGEN, transactions of the Natural History Society of, i. 297; mode of examination in the Academy of Surgery at, 399; report on the cow pox at, ix. 462.

COPHOSIS, case of, xxv. 510.

COPPENS, Mr. his method of decomposing sea-salt, i. 301.

COPPER, the colouring part of blood dissolves, vi. 470; changes produced in, by hot lava, 570; chemical analysis of muriated, ix. 475; effects of potash on, xiv. 382; precipitated from its solution in sulphuric acid, xv. 582; the supposed cause of the poison of fish, xx. 415; xxv. 390; a bank so called, xx. 466; gives a red colour to glass, xxv. 363; purgative quality of the phosphate of, 456; analysis of the sulphate of, xxx. 345.

COPPER-GLANCE, description and analysis of a variety of the, xxi. 262.

COQUART, M. his observations on the effects of lightning and electricity in gonorrhœa, vi. 277.

CORALS, description of some fossil, xxviii. 239.

CORAY, a modern Greek, his version of the works of Hippocrates, i. 89.

CORDET, Charlotte, anecdote concerning, i. 52.

————, M. his analysis of coffee, xx. 384; on the unwholesomeness of tea, xxiv. 189; observations in reply to, 265.

CORDILLERAS, in South America, description of that chain of mountains, xiv. 189; xx. 11.

CORDUS, Valerius, memoirs of, xxii. 270; account of his pharmacopœia, 271.

CORIANDER SEEDS, stomachic and carminative virtues of, xii. 369.

CORK, analysis of, iii. 89; on the acid produced by, vi. 276.

————, (City of,) mortality among the troops there, i. 228; account of the influenza at, x. 523; remarks on the contagion there, 525; account of the hospitals at, xv. 357; production of furze near, xix. 75.

CORKINDALE, Dr. on a case of tic douloureux, xx. 180.

CORN, extraordinary production of Indian, i. 47; method of extracting sugar from Indian, iv. 245; examination of the spikes of, 246; on the earthy particles in, vi. 568; chemical experiments on the saccharine matter in Indian, xx. 482.

CORNAX, Matthew, an old medical writer, account of his works, ii. 457.

CORNEA, description of an instrument for dividing the, i. 333; case of tumour on the, v. 241; successful case of opacity of the, viii. 398; treatment of ulcers of the, x. 563; its incision censured, ibid.; anatomical account of the, xi. 88; on the nebula of the, xvii. 79; danger of puncturing the, 81; on the changes produced in the transparency of the, 193; new membrane discovered in the, 283; correction respecting it, 527, on the mortification of the, xx. 174; on excrescences and pustules of the, 186; on abscesses of the, ibid.; on the ossification of the, 187; on the formation of specks in the, 188; its appearances after death, 189; on the sensibility to light in the inflamed, 472; method of making an incision in the, xxi. 146; case of ulcerated, xxiii. 57; observations on ulcers of the, 78; case of tumour of the, 125; on the projection of the, xxiv. 161; on puncturing the, xxvi. 535; on the too convex form of the, xxvii. 114; case of erythema mercuriale, accompanied by an affection of the, 420; description of the membranes of the, xxx. 93; on the puncturation of the, xxxi. 508.

CORNELLI, Mr. on the disadvantages of the common method of evaporating vinegar, i. 80.

CORNISH, James, his case of croup, xxix. 268; case of death occasioned by laudanum, xxxi. 193; his case of epidemic convulsion, 373; observations on the case, 564; his reply, xxxii. 32.

CORNS, the round leaved sundew a remedy for, xiv. 64; the red spurge a cure for, xvi. 75.

CORNWALL, salubrity of the climate of, xi. 284; analysis of a new mineral found in, xvi. 95; furze used as fuel in, xix. 75; vein of silver found in, xxvii. 169; mineralogical observations on, xxviii. 77; remarkable effects of fanaticism in, xxxi. 373; observations on that case, 564; reply to the observations, xxxii. 32.

CORNWALLIS, Marquis, address to him on the subject of vaccination, vii. 201.

CORPORA LUTEA, remarks on the, v. 587.

CORPULENCE, remarks on, xxv. 449; cases of, 450; on its frequency in England, 451; reduced by diet, 452.

CORYPHENE, a fossil fish of that genus, discovered in France, x. 480.

CORROSIVE SUBLIMATE, advantage of treating venereal diseases with, ii. 469; effects of alkalis in counteracting the poison of, iii. 535; recommended in amaurosis, iv. 184; useful as a collyrium, vii. 215; experiments with, 548; analysis of, 550; how converted into calomel, 551; useful in cutaneous complaints, viii. 113; on the preparation of it, ix. 81; new method of preparing, xi. 340; on the change of its name, xiii. 222; its value in venereal cases, 551; case of uncommon disease produced by, xviii 347; its efficacy in an enlargement of the knee-joint, xxii. 515; case of poison by, xxvi. 115; its effects on different animals, xxviii. 126; case of poison by the external application of, 223.

CORSE, Mr. his observations on the copulation of elephants, v. 588.

CORSHAM, Wilts, account of the influenza at, x. 115.

CORSICA, introduction of exotic plants into, vii. 479.

CORTEX CARIBÆUS, its effects in rheumatic complaints, vi. 376.

CORTEX SALICIS LATIFOLIÆ, cautions respecting the right choice of, xi. 400; on the medicinal virtues of, xii. 372; substitute for cinchona, 373.

CORUNNA, on the diseases of the troops in the expedition to, xxii. 428; xxiii. 156, 517.

CORVISSART, M. account of his Journal de Medicine, vi. 174; on a case of fistula in the stomach, viii. 361; on organic diseases, xxiii. 277; on the diseases of the heart, xxx. 141.

COSME, Frere, his operation for the stone, iii. 195.

COSMETICS, one much used by the Turkish ladies, extracted from Balm of Gilead, i. 34; one extracted from the nymphea helumbo, iii. 273; the round-leaved sundew recommended as an excellent one, xiv. 62; Solomon's Seal used for, 65; the arum or wake-robin, good as, xvii. 73; the fumitory a beneficial one, xix. 72.

COSSIGNY, M. on the juice of the papaya, ix. 79.

COSTIVENESS, carbon a remedy for habitual, i. 279; farther observations on that remedy, 393; to be removed in gout, xii. 563; singular case of, xv. 100; observations on the causes of, 102; caution to be used in treating, 336; on the benefit of opium in, 337; cured by olive oil, xix. 51; benefit of assafœtida in, xxi. 477; remarkable case of obstinate, 480.

COTTA, Dr. John, account of his work on quackery, xviii. 3.

COTTON, effect of mordicants in dying it red, i. 165; on ascertaining adulterated, ii. 83; the plague said to have been conveyed in bales of, vii 311; ix. 503; that point controverted by experience, xxvii. 272; improvement in the dying of, xxii. 525; effects of swallowing a skein of, xxiii. 431; method of taking iron-moulds out of, xxx. 84.

———, Dr. of St. Alban's, anecdotes of, xiv. 370.

COTYLEDON CRENATA, observations on the, xxvii. 260; on that of grasses, xxix. 510; change in the leaves of, 511.

COUCHING, that operation preferred in cataract to extracting, x. 370; method of performing it, 371; its safety, ibid.; on the needle used for, xv. 103; advantage of the seton after, xvi. 208; case in which it was successful, xix. 102; observations on the practice, 104; causes of the failures in the operation of, xx. 174; method of operating for it in infants, xxvii. 161.

COUGH, efficacy of digitalis in obstinate, i. 123; holding the breath recommended in, 260; on the use of yeast in, iii. 105; efficacy of digitalis in obstinate, iv. 537; necessity of attending to the early stage of, v. 16; use of opium in obstinate, vii. 306; relieved by opium, ix. 270; advantage of bleeding in, 275; veronica recommended in, xi. 371; cases of obstinate, xv. 223; not always an indication of catarrh, 463; consequences of a neglected, xvii. 484; case of violent, by swallowing a pea, xviii. 54; an excellent decoction for the cure of, 458; milkwort good for catarrhous, xix. 73; virtues of colt's foot in relieving, 359; case of violent and obstinate, cured by preparation of iron, xxii. 70; on winter, xxiii. 150; on that of the hydrothorax, 276; observations on Dr. Buxton's treatment of, xxx. 49.

COUNTENANCE, on the human, ix. 480; an index of the mind, xix. 2.

COUP DE SOLEIL, impropriety of the term, vii. 215; difficulty in the method of treating, xiii. 487; cases of recovery from, xxiii. 196, 198; fatal case of, 198.

COUPER, Dr. observations on his idea of generation, v. 404.

COURTOIS, M. his discovery of a new gas, xxxi. 255.

COWARD, Dr. his notice of Mayow, i. 221.

COWHAGE, its efficacy in worms, iv. 567; method of preparing it, xiii. 87; advice in the use of it, 88; observations on its efficacy, xxviii. 413; xxix. 335.

COWLEY, on his poetic representation of the virtues of plants, xxvi. 295.

———, J. his account of a dislocation of the jaw, iv. 503.

COW-POX, history of the discovery of the, i. 1; symptoms in the cow, 2; and in the human subject, ibid.; taken from the grease in the heels of a horse, 4; new light thrown on the animal economy by, 5; permanent security against small-pox, 7; its origin in the grease of the horse questioned, 10; a gentleman who had it twice, 11; Dr. Sims's doubts on its safety, 12; progress of it in London, 113; on the origin of the, 114; case of, by Mr. Walker, 117; noticed in France, 199; prize question at Guy's Hospital on, 202; history of the inoculation for the, 217; Dr. Sims in explanation on, 230; experiments to ascertain the origin of, 309; Dr. Jenner's further observations on, 313; on the spurious, 314, 323; cases of inoculated, 318; its security doubted, 322; Dr. Woodville's reports on the, 323, 417; cases of inoculation, by Dr. Redfearn, ii. 23; remarks on the, 25; facts relating to the, 97; cases of, 106, 134; small-pox after the, 107; statement of its progress, 213; trials to determine its security, 219; introduced at Vienna, 225; successful in Scotland, ibid.; extracts of letters on, 310; Dr. Croft on the, 391; Mr. Abernethy on, 401; Rev. Mr. Holt on his successful practice of, 402; on the eruptions produced by, iii. 92, 97; on the appearance of pustules in, 101; institution established for the inoculation of the, 175; cases of, by Mr. Stewart, 234; Dr. Remmett in favour of, 237; Dr. Huggan on the efficacy of, 241, 344; Mr. Fosbrooke in answer to Dr. Pearson, on eruptions in, 247; Dr. Denman on the advantage of, 292; experimental observations on, 294; Mr. Shorter on its efficacy, 348; Dr. Jenner's letter, in answer, 351; his continuation of facts and observations, 381; on the present state of, 399; on the report of fatality attending it, 411; Rev. Mr. Finch on his practice of it, 415; Mr. Dunning on the benefits of, 436; queries concerning, 437; its success in Hanover, 471; Dr. Jenner's answers to queries on, 502; Dr. Barry's observations on, 503; Dr. Trotter on the use of, 525; Dr. Denman on its security, iv. 2; Earl of Derby on the inoculation of his children, 3; Mr. Cooke in retractation on, 20; success of it in Kent, 21; beneficial to population, 41; address on the advantages of, 69; practised at Paris, 88; Dr. Juncker's letter to Dr. Pearson on the advantages of, 141; classical definition of, 146; successful at Cork, ibid.; on the safety of, 171; medical testimonies in London in favour of, 187; on the introduction of the practice at Paris, 251; Dr. Woodville's observations on, 254; reflections on the, 257; introduced into the navy, 307; a sure preventive of small-pox, 337; successful cases, 338; introduced with effect at Montrose, 340; on a case of small-pox after, 343; series of facts respecting the, 381; conveyed to the Mediterranean, 386; cases of, by Mr. Custance, 421; progress of it in Ireland, 425; in Yorkshire, 429; comparative statement of facts and observations relative to the, 463; on preserving the matter of, 470; communicated to America, 471; on the danger of inoculating with purulent fluids, 488; caution respecting, 489; cases of the inoculation of, by Mr. Sarjeant, 505; on its superiority over variolous inoculation, 518; it success at Gibraltar, 544; prospect of exterminating the small-pox by, 563; case of, in the family of Dr. Lettsom, 567; testimonial in favour of, at Leeds, Durham, and Chester, 570; on its practice in the Small Pox Hospital, v. 6; does not hybridise with small-pox, 8; cautions respecting the inoculation of, 17; necessity of inspecting patients in, 22; early knowledge of it in Ireland, 24; on the want of care in taking matter of, 25; observations in irregular cases of, 26; on tearing a vesicle in, 27; true character of, 28; on the comparative mildness of, 32; the air not contaminated by, 33; its progress in the navy, 74; on supposed failures of the, 86; report of the committee at Paris on, 99; resolutions of the medical practitioners at Bradford, on the, 102; Dr. Harrison on a case of, 108; Rev. Mr. Gale's account of his practice in, 111; counties in which it is known, 112; sources of error in, 134; on the origin of the, 136; a supposed failure in, 151; cases of its success, 152; directions for preserving the matter of, 154; introduced at Plymouth, 156; its triumph at Vienna, 157, 351; small-pox renders persons insusceptible of, 160; Mr. Maddock's cases of, 161; state of it at Constantinople, 243; in the Mediterranean, 263, 315; on cases of a spurious kind of, 281; practical observations on, 292; on the nature and effects of, 293; on the preservation of the matter of, 338; cases of, 340, 341; introduced into the Venetian states, 352; its success at Chatham, 354; letter to the people of Paris on, 356; remarks on the discovery, 359; concise view of the most important facts in the, 383; evidences on the utility of the, 384; essay upon the inoculated, 386; Rev. Mr. Jenner on cases of, 401; cases of its security against inoculated small-pox, 430; medal for the discovery of, 432; Mr. Grose's cases of, 452; on the preventive power of, 455; history of the origin of, 505; its success abroad, 543; cases of its success, 547; history of the progress of, 554; observations on its efficacy, vi. 5; on its introduction at Montrose, 36; characteristics of it, 40; on irregularity in, 45; observations on, by Dr. Winterbottom, 46; its progress in America, 64; dogs inoculated with the, 95; successful in Sicily, ibid.; Lord Berkeley on the, 105; Essex resolutions on the, 113; children inoculated for it at the Birmingham Asylum, 114; address to the poor on, 115; on spurious kinds of, 117, 121; case of erysipelas following it, 131; cases of its preventive power, 133; on the failure of, 218; advice respecting, 219; observations on a phenomenon in, 220; case and remarks on, 236; London testimonial in favour of, 239; its progress on the Continent, 280; its effects on dogs, 314; on the necessity of caution in the practice of, 323, 325; on the spurious, 326; on the difficulty of preserving the matter of, 327; observations on, in America, 330; known in the sixth century, 379; on the denomination and description of, 386; chemical analysis of the virus of, 395; proposal on the discovery of, 422; case of inflammation after, 423; on the supposed antiquity of it, 486; on pretended failures in, 487; proved to be a preventive of small-pox, 488; opposed in Germany, 571; on misrepresentations respecting the, vii. 2; necessity of zeal in promoting it, 3; beneficial effects of it proved, 7; on distinguishing the genuine, 9; on the success of, 11; its progress in Russia, 109, 292; and in Austria, 110; historical view of the, 168; practical observations on the, 169; its progress in Spain, 171; indigenous in Lombardy, 172; on the origin of, 175; manner of procuring pus for inoculation, 178; on the spurious, 182; its appearance on the udder of the cow in Lombardy, 184; observations and experiments upon the, 185; advantages of it, 191; French address on the, 201; on the spurious vesicle in, 203; on a supposed case of fatal, 250; communications from Rotterdam on the subject of, 251; its reception in Turkey, 292; observations on the practice of it, 294; proposal for extending, 378; Suffolk address on, 386; report of the Committee of the House of Commons on, 485; resolutions of the Colchester Medical Society on, 528, its success in Norfolk, 540; evidence before the committee on the, viii. 17, 141; successful in Staffordshire, 26; at Constantinople, 95; on the reward for the discovery, 164; observations on the inoculation of, 165; attempt to prevent, 169; on supposed unsuccessful cases of, ibid.; facts urged against the practice, 182; facts decisive in favour of it, 185; account of its progress in Austria, 193; encouraged by the King of Prussia, 195; remarkable case of, 197; on alleged unsuccessful cases of, 198; practice of at Bath, 208; practical observations on the inoculation of the, 373; new method of preserving the matter of, 374; test of constitutional affection, 375; researches into the early history of, 475; letter from the empress dowager of Russia on, 480; its success at Boston, in America, 543; at Liverpool and the neighbourhood, 546; case of alleged failure, 547; address of the vaccine pock institution, on the, 565; practised at the Bloomsbury Dispensary, 568; nosological description of the, ibid; its progress in Spain and Italy, 577; cases where it could not be communicated, ix. 38; directions for the inoculation of, 65; analysis and experiments on the matter of, 67; its progress on the Continent, 128; London Society for extending the, 192; report of the Small-Pox Hospital committee on, 194; successful in the Southern States of America, 196; address from the Finsbury Dispensary on, 274; patronized by their Majesties, 292; necessity of care in, 296; superiority of tooth-picks for conveying, 297; successful in India, 299; introduced at Madeira, 310; on the advantages of, 357; objections to, 358; cases of, 363; suspended by Varicella, 370; practised at the Finsbury Dispensary, 373; at Ceylon, 391, 457; plan and instrument for preserving the matter of, 397; on the objections to it, 413; interest excited by it in Italy, 435; success of it in the East, 450; tried upon sheep, 455; Copenhagen report on, 462; glass the best means of conveying it, 464; increase of the practice, 467; observations on Dr. Pearson's claims to improvement in, 481; on the danger of, 484; on convulsions following, 532; Bombay medical report on the, 534; letter from the Earl of Hardwicke on, 540; answer to his lordship, 541; table shewing the advantages of, 543; striking instance of small-pox superseded by, 544; on quills for preserving the virus of, x. 40, 41; fully established in India, 93; its origin ascertained, ibid.; on instruments for preserving the matter of, 160; report of the Vaccine Institution on the state of it, in 1803, 187; plan of lectures on the, 190; state of it at the Small-Pox Hospital, 191; sheep inoculated with, 192; letter from Dr. De Carro on, 242; comparative degrees of mildness in, 322; cases of supposed failure in, 421; introduced at Barbadoes, 424; a security against the plague, 474; its progress at Jamaica, 544; at Quebec, 546; in Greece and Turkey, 547; on the activity of the matter in hot climates, xi. 39; negroes remarkably susceptible to, 40; case in which it did not appear till the forty-sixth day, ibid.; its preventive powers proved in a child where the mother had the small-pox, 41; cases of its resistance to variolous infection, 131; its infallibility determined, 251; a remedy against atrophia, 252; miscellaneous remarks on, 317; parallel between the small-pox and the, 323; a specific against that disease, 325; case of its being succeeded by an eruption on the labia pudendi, 385; on the blessings of, 433; examination of supposed failures in, 505; xii. 44; caution respecting the treatment of children in, xi. 552; meeting of the society for promoting, 572; state of the inoculation for it, 573; resolutions of the county of Sussex in favour of, xii. 10; its success in Scotland, 11; reply to Dr. Walker on, 49; cases of small-pox subsequent to, 79; notice on the, 90; its effects on sheep, 93; rendered ineffectual by herpetic affections, 97; on modifications of the vesicle of the, 98; its rapid progress in Asia, 122; foul conduct of its adversaries, 165; on alleged cases of failure, 167; advantages of the discovery, 168; answers to Mr. Goldson on, 169, 181; on encouraging objections to, 195; its efficacy, 205, 207, 209; on supposed failures at Portsmouth, 211; on the matter of, 241; cases of inoculation and re-inoculation of, 263; experiments to ascertain its security, 264; observations on the cases of Mr. Goldson, 298, 331; Dr. Walker's reply to Dr. Adams on, 332; on the proper term for, 335; observations of the French society for the promotion of vaccination, on Goldson's cases, 348; on the vaccine vesicle, 356; on eruptions succeeding, 358; on a supposed case of, 360; its effects in Ceylon, 383; placed under a director-general in Italy, ibid.; a case of small-pox after it, said to have occurred in Fulwood's Rents, 384; experience of its efficacy after much doubt, by Mr. Trye, 395; observation on its safety, 398; on the propriety of removing the scab in, 439; observations on the proper term for, 440; on the case in Fulwood's Rents, 441, 534; address to the inhabitants of Malta on the, 443; on a case at Kensington, 446, 529, 539; analysis of Dr. De Carro's historical work on the subject, 447; adopted at Athens, 448; encouraged with zeal by the Armenians, 451; remarks on a case of, 467; experiments proving it a security against small-pox, 473; plan of the Sussex Institution for promoting, 477; new term proposed for, 517; observations on Dr. Rollo's cases of, 531; on eruptions after, 536; on extending it to New South Wales, 537; address to the governor of Minorca on, 540; observations on spurious and degenerated, 542; chicken-pox mistaken for, 543; result of inquiries concerning its progress in Britain, 554; report on the Fulwood's Rents case, 573; attended with a secondary eruption, xiii. 1; candour recommended to the parties engaged in the discussion, 3; general in the Highlands, 37; case of re-inoculated, 38; cases in Philadelphia, 40; unsuccessful attempts to inoculate, 42, 43; cases of re-inoculation, 44; on the blue pocks in, 47; excited with a scab nine months old, 48; observations on a second inoculation of, 50, 51; its success at Aberdeen, 52; observations on the report of the Fulwood's Rents cases, 53; caution necessary with respect to the lancet in, 54; cases of it at Newcastle upon Tyne, 57, 65; violent inflammation in, 62; on the terms used for, 65; resolutions of the Plymouth Society on the, 91; its establishment at Ceylon, 122; observations on the inoculation for, 165; its increasing interest, 168; not an eruptive disease, ibid.; on miliary eruptions after, 171, 245; resolutions of the Somerset Jennerian Society, 187; statement of persons inoculated for it in London, 188; on pretended failures of, 246; children inoculated who had herpetic eruptions, 250; small-pox after, 251, 268; rapid progress of it in India, 283, 336, 381; abstract of persons vaccinated there, 284; at Edinburgh, 286; on the objections to it, 332; cases of its success in Yorkshire, 336; state of it in Ireland, 379, 380; on the slough in, 399; on herpetic eruptions in, 415; cases of vaccination at Plymouth, 417; supported by the French government, 419; regulations adopted in France respecting the, 426; on cases of incomplete, 455; on preserving the vesicle in, 456; on spurious cases of, 457; an attempt to prove its identity with scrofula, 465; Dr. Moseley's treatise on, 466; examination of the evidence on, 468; its progress in Turkey, 479; cases of inoculation with that and the small-pox, 517; on cutaneous eruptions impeding the action of, 537; case of herpes in, 541; impropriety of breaking down pustules in, 542; prejudices of the Hindoos against it, 569; on cases of small-pox succeeding, 572; remarkable history of the practice in the East, 574; cases of small-pox after, xiv. 5; eruptions ascribed to, 6; irregularity in the process occasions tinea capitis, ibid.; cases of small-pox after, 21, 22, its protective power known in Holstein, 28; antivariolous power of the, 48; causes of failure in the, 49; herpetic eruptions occasion it to be incomplete, 54; on secondary cases of, 55; repeated failures in the same subject, 57; resists small-pox, 58; address to the medical practitioners in Ireland on, 84; queries connected with the, 95; on miliary eruptions in, 145; observations on attempts to depreciate the value of, 174; its success in Prussia, 233; letter from the Earl of Westmeath on, 256; address to the people of Norwich on, 277; on the preservation of the matter of, 288; observations on cases of it at Plymouth, 308; meeting of the Sussex Institution for promoting, 346; address of the Royal Somerset Society on, 348; depressed by variolous inoculation at the Small-Pox Hospital, 360; letter of Mr. Birch on the failures of, 376; answers to all the objections made to, 377; children re-inoculated with small-pox after, 402; anecdote of its early history, 407; number of persons vaccinated in the East, 409; mistakes corrected respecting, 410; case of eruptions ascribed to, 411; indiscretion in some of its friends, 433; its security, 435; its reception in Ireland, 437; attempt to prove it the small-pox translated to the cow, 480; cases of re-inoculation at the Foundling Hospital, 506; anecdote of casual, 510; cutaneous diseases erroneously ascribed to the, 526; generally adopted in Hanover, 535; cases of its failure, 536; flaming invective against it, 565; letter from the Earl of Egremont on, 574; on its supposed identity with small-pox, xv. 48; cases of re-inoculation of, 56; on pretended failures in, 57; cases of children submitted to test in, 58; cutaneous diseases removed by it, 61; address to the inhabitants of Yarmouth on, 92; report of the Royal Jennerian Society in 1806, 107; its progress and success at Newcastle, 135; observations on pretended deformities from, 157, 167; case of small-pox after, 169; radical difference between that and the small-pox, 219; observations on Dr. Rowley's cases, 231; instances of its efficacy in resisting small-pox, 233; on rupturing the vaccine vesicle in, 238; on its preventive powers, 246; institution at Nottingham for promoting 251, 426; case of its prophylactic power, 257; on the disputes occasioned by it, 284; letters to Rowley in defence of it, 286; vindication of the, 290; comparative merits of that and the small-pox inoculation, 301; observations on Birch's queries respecting, 304; cases, 305; remarks on cases of spurious, 308; account of it in Canada, 311; among the North American Indians, 313; in France, 314; in Dublin, 319; on the preventive effects of the true, 350; derived from the grease in the horse, 382; its progress in Ragusa, 390; on the distinction between vesicle and pustule in, 429; on the varieties in the, 431; on the use of ivory lancets for inoculating, 516; vaccinators preferable, 517; inoculation with the grease of horses, ibid.; sheep inoculated with it, ibid.; history of vaccination in India, 518; on the eruptions after, 520; introduced in the Isle of France, 523; at the Cape of Good Hope, 524; prejudices excited against it in England, 529; resolutions of the Liverpool Society for promoting, 536; on the doubtful origin of, 539; case of inoculation resisted till the twenty-ninth time, 545; on breaking the vesicle, 546; on the pus in the middle of the pock, 547; sketch of a new theory of the, 571; itch ascribed to, 579; caution to be used respecting, 580; traced from the heel of a horse, 582; on the endeavours to depreciate, xvi. 25; forgeries against, 27; Dr. Beddoes in favour of, 43; testimonial of the Colleges of Physicians and Surgeons in its recommendation, 44; case of small-pox after, 67; on the spurious kind of, 69; cautions necessary to insure its success, 71; case of inflammation after it, 73; vindication of the practice, 82; Chinese treatise on it, 96; cases of supposed small-pox after, 110; its origin in the horse asserted, 122; a cow inoculated with equine matter, 129; cases observed of the origin of the disease, 131; report of the Nottingham Institution on, 132; on a test of the disease, 138; comparative view of the inoculated cow-pox and small-pox, 139; on its advantages, 181; imposture attempted with regard to the, 188; known among the Mogols, 192; on the equine origin of, 215, 239; natural in the cow, 216; cases of it,* 218; advantage of opposition to it, 220; remarkable cases proving its origin, 239; the udder of a cow inoculated, 241; a disease resembling the, 246; on herpes observed during the, 247; cases of re-inoculation of, 260; reward offered for an unsuccessful case of, 284; on the state of the controversy, 315; on the criterion of the true, 317; danger of cutting up a vesicle in, 323; instructions for the practice of inoculation, 325; cases of supposed failure, 326; equine origin of it questioned, 328; observations on the proper appellations for the, 334; characteristics and effects of the perfect, 372; progress of the vesicle, 373; on the imperfect, 375; observations on cases, 379; account of a case where it was unsuccessful till after repeated attempts, 436; ascribed to idiosyncrasy in the patient, 437; on the causes of scepticism concerning, 441; on the matter of inoculation, 442; farther proofs of its origin in the heel of the horse, 443; the spurious sort mistaken for the real, 447; on irregular vesicles, 448; not the cause of cutaneous eruptions, 449; on cases of supposed failure in, 460; explanation of a case of small-pox after, 473; error of inoculating with dry matter, 529; letters of Mr. Fermor on the discovery, 541; letter from Dr. Wall on, 546; successful in China, 573; observations on the practice of it, 574; cases at a farm near Berkeley, xvii. 11; voyage to propagate it in the Spanish colonies, 12; cases of its efficacy, 25; its success in Germany, 57; in America, 62; in Italy, 63; pustulous discharge of matter after, 95; on tests for the, 136; case of inflammation after, 156; the practice vindicated, 175; Danish report on the, 190; case of small-pox after, 233; not properly understood, 240; a mission sent to propagate it in Siberia and Tartary, 248; its success in Buckinghamshire, ibid.; its rapid progress in Denmark, 295; cases of, 312; cases of supposed failure of, 330, 365; observations on it, by Mr. Burns, 471; report of the Foundling Hospital on the, 488; case of small-pox after, 514; account of its success in Ceylon, 517; and on the continent of India, 540; historical retrospect of, xviii. 23; contrasted with small-pox, 26; account of Balmis's voyage to disseminate the, 37; abridgment of papers on, 70; report of the College of Physicians of London on, 97; of the College of Physicians in Ireland on, 104; of the physicians of Edinburgh on, 105; of the London College of Surgeons on, 106; of the College of Surgeons at Edinburgh on, 109; of the College of Surgeons of Ireland on, 110; false alarm excited respecting its safety, 136; refutation of erroneous statements concerning the, 139; its reception at the Cape of Good Hope, 147; reply to Mr. Marson on, 150; resolutions of the medical gentlemen at Worcester, in favour of, 190; its success in Arabia, 191; opposition to it, 238; on cases of pretended failure in, 316; unfair practices of its adversaries, 317; ignorantly called a new theory, 311; cure of tinea capitis by, 340; unaccountable prejudices against it, 566; legislative interference necessary to enforce it, 567, 572; its beneficial influence on the human frame, xix. 151; history of failures at Ringwood, 193; report on the Ringwood failures, 327; on the violence of the controversy respecting, 377; on the secondary vesicles in, 380; on the practice of, 419; report of the Dublin Institution for promoting the, 452; on the supposed failures at Ringwood, 557; historical retrospect of, xx. 21; methods of preserving the virus, 23; cases of its failure, 61, 237; effects of a general inoculation for the small-pox on it, 259; case of secondary 333; striking instance of its salutary effects, 334; its success at Groningen, 336; on the cellular structure of the vesicle in, 337; inquiry into the unfavourable reports at Cambridge on the subject of, 363; observations on the eruptions called spurious, 534; remarkable case of, 535; resolutions of the Colchester Medical Society, on the, 565; on the want of candour in the controversy, xxi. 121; national establishment for the, 170; success in the Brazils, 173; account of an eruptive disease after, 250; coincidence between it and small-pox, xxii. 87; unsuccessful cases of, 88; test of security for persons who have passed it, 173; advantage of, 343; on the state of it at Madeira, xxiii. 94; causes of its retardation in England, 111; on the alleged cases of failure in, 112; circular letter on the subject, from the College of Physicians, 114; unfounded prejudices against it, 123, 172; obstinacy of the Scotch in regard to, 258; scientific importance of the discovery, xxiv. 32; account of publications on, 33; its origin and changes, 47; progress of it in London, 86; on the gratuitous inoculation of, 88; opposition to it in Scotland, 163; description of the genuine vesicle, 479; on irregular vesicles, 481; on constitutional symptoms, 483; report of the Dublin Institution on, 518; its success in the Foundling Hospital at Dublin, 522; report of the Nottingham Institution on, 523; system adopted in France for promoting, xxv. 181; address to the nation on, 278; its success at Liverpool, 304; case of small-pox subsequent to, 390; progress of it at Glasgow, 458; reports on the state of it at Edinburgh and Ceylon, 460; cases of small-pox after, xxvi. 82, 177, 219, 234, 249; observations on its progress, 390, 398; report on failures in, 416; security of it established, 417; experiments on, 418; result of eleven years practice in, 488; introduced into Iceland, xxvii. 122; cases of small-pox after, 164; case of chicken-pox occurring with, 171; received gladly by the Indians of North America, 383; on the occurrence of small-pox after, 422; its success at Jamaica, xxviii. 78; cases of small-pox after inoculation of the, 111; proposal for rendering it universal, 159; its effect in reducing mortality by the small-pox, 180; report on the progress of the practice, 190; remarkable case of secondary appearance in the same patient, 382; observation on the case, 452; successful in exterminating the small-pox in Ceylon, xxix. 73, 155; cases of small-pox subsequent to, 281; xxx. 33; testimony of the Royal College of Surgeons in favour of, 86; report of the National Vaccine Establishment on, 113; progress of it in India, 216; address of that establishment to medical practitioners, 257; resolutions in Gloucestershire on, ibid.; French report on the benefits of, 519; Norwich report on, xxxi. 136; improbability of measles being influenced by, 273; method of preserving the matter of, 432; its success at Bagdad, 520; on the National Establishment of, xxxii. 107; on the altered specific powers of, 180; supposed modification of the small-pox, 211; on its security, ibid.; on the recurrence of small-pox with, 213; on the inoculation of the, 214; state of the practice in Sweden, 250; its effect on hooping-cough, 256; advantages of the discovery, 289; rules for the inoculation, 290; agency for it in America, 347; observations on small-pox after, 478; on the nature of the fluid in, xxxiii. 17; caution to be used in the practice of inoculation, 18; regulations respecting the practice, 69; report of the Small-Pox Hospital on the, 250; observations on the failures of, 257; on the rejection of the bill for encouraging it, 113; report of the National Vaccine Establishment for 1814, xxxiv. 166; analogy observed between that and the small-pox, 295, 387; on the occurrence of mitigated variola after, 452. See *Vaccination*.

COWS, on the diseases and habits of, i. 2; symptoms of the vaccine pox in the, 3; on the advantage to consumptive persons of living with, ii. 415; the beet an excellent winter food for, iii. 9; on the supposed good effects of living with, 14; analysis of the liquor amnii of, iv. 185; vaccine matter obtained from, 255; experiments with the grease of a horse on, 466; case of a man gored by one, xii. 270; one sucked by a snake, xiii. 287; observations of the Hindoos on disorders in, 575; history of the inoculation of one, xiv. 29; attempts to inoculate them with variolous matter, xv. 49; affected by the grease of horses, 582; on the pulse of, xvii. 93; the heat and exhalation of them beneficial to consumptive persons, xxv. 343; recovery of one that was bitten by a mad dog, xxvi. 206; on the period of gestation in, xxviii. 432.

COX, D. his account of a singular affection of the eyelids, vi. 393; account of his medical compendium for families, xxi. 167.

COXE, Dr. Redman, his experiments on the opium obtained from the lettuce, vi. 396; his observations on the cow-pox, viii. 565; reply to, xi. 318; his cases of re-inoculation, xiii. 40, 47; on bronchocele, 47; on a case of chorea sancti viti, with a new theory of that disease, 401; remarkable history of a case of tetanus, xiv. 129; his theory of chorea controverted, xv. 119, 135; his case of burn, xvi. 9; account of his American Dispensatory, 570; xviii. 41; his reply to Dr. Patterson on chorea, xviii. 213; history of a case of extensive burn treated with turpentine, 304; on a case of tinea capitis cured by vaccination, 340; case of tetanus treated ineffectually with cantharides, 436; account of an extraordinary cure of a wound in the intestines, 501.

CRAB-TREE, juice of the fruit good for sprains, xvi. 170.

CRADLE, description of one for fractured legs, xxxiii. 170.

CRAIB, Mrs. particulars of a supposed case of cancer in the breasts of, iv. 546; farther account of her case, v. 76, 188.

CRAIG PHADRIC, a vitrified fort in the shire of Inverness, xxviii. 241.

CRAMP, in the stomach, description of two species of, i. 49; relieved by emetics, 50; diet to be used in, 51; magistery of bismuth efficacious in, 504; on the good effect of ether and tinct. opii in, iv. 444; pepper used externally in cases of, xii. 471; benefit of the oxidule of bismuth in, xxiii. 501.

CRAMPTON, Philip, his description of an organ in the eyes of birds, xxix. 481; his operation for popliteal aneurism, xxxiii. 158, 516.

CRANBERRIES, botanical description and utility of, xiv. 174.

CRANE, Dr. his observations on a case of impregnation, xiv. 416.

CRANIOLOGY, concise account of Dr. Gall's doctrine of, xiv. 327; general view of his system, xv. 201, 203; organs of his system, 213; observations on, xviii. 38; decline of that system, xx. 17; report of a memoir concerning, xxi. 149; observations on it, xxiv. 3; statement of the particulars of that doctrine as taught by Gall and Spurzheim, xxxiii. 228; proofs in favour of it, 252; further account of the system, 485; vindication of it, xxxiv. 309.

CRANIUM, case of the dura mater detached from the, i. 328; case of fracture of the, iv. 346; another case, 513; unusual circumstances in a fracture of the, v. 255; case of fracture in the upper part of the, vii. 53; on effusions under the, 216; on the structure of the, viii. 466; fissure of the cranium, attended with apparent extravasation, x. 244; observations on fractures of the, 370; instance of the fracture of a remarkably thin one, xi. 36; another case, xiii. 295; two cases, with depression, 526; on recent depressions of the, xiv. 161; on the structure of the, 98, 529; case of epilepsy occasioned by an injury to the, xvi. 473; on malconformations of the, xxi. 282; on the capacity of the, 334; discovery of some monstrous fossile ones, xxiv. 87, 100; case of fracture, where the dura and pia mater were lacerated, xxviii. 154; caries of the bones of the, xxix. 177; case of extensiv fracture of the, xxxi. 485.

CRATAEGUS OXYACANTHA, account of the caterpillar found on hedges of the, xxi. 92.

CRAWFORD, Dr. his theory of heat, i. 162; his observations on vital air, 371; on the nature of cancerous matter, 430, 431; on the inspiration of oxygen gas, viii. 500; his experiment on muriatic acid, xi. 291; his theory invalidated, xiii. 373; vindication of his opinion on cancerous matter, xiv. 467; inaccuracy of his experiments, xix. 336; objections to his theory of animal heat, xxi. 67, 70; on the deterioration of air by respiration, xxiii. 310; his experiments on caloric, xxvii. 11; observations on his theory, xxix. 150; objections to his hypothesis on animal heat, xxxiii. 113, 120.

CRAWFORD, Dr. John, his case of hepatic affection, xxiii. 226; his remarks on quarantines, xxiv. 23; a great champion for the animalcular system of diseases, 24; on his translation of Haller, 38.

————, Nathan, on the efficacy of poke root in hydrophobia, xvii. 294.

CREAGH, P. T. his cases of fractured cranium, xxviii. 154.

CREASER, Thomas, his evidences of the utility of vaccine inoculation, v. 384; his observations on spurious vaccine, vi. 326; chemical analysis of vaccine virus, 394; on the efficacy of opiate friction, viii. 16; his practice of vaccination, 208; his observations on Dr. Pearson's evidence, ix. 286; his account of the influenza at Bath, x. 114; on the Jennerian Institution in that city, xiii. 186; on spurious pustules, xv. 308; his report of the Royal Somerset Jennerian Society, 309; correspondence with him on the ravages of the small-pox in Somersetshire, xvi. 23; reply to his remarks by Dr. Wood, 64; on his definition of a spurious pustule, 69.

CREDITON, account of the influenza at, x. 311; remarkable cases of vaccination, xxxiv. 295.

CREOLES, not liable to be infected by the pestilential fever, xxi. 248.

CRESSES, experiments made with garden, iv. 84; virtues of water, xviii. 274.

CRETINISM, observations on the appearance and causes of, v. 175; accompanied with mental imbecility, xvi. 208; general view of its appearances in Savoy and Switzerland, xxii. 9; account of different works published on that subject, 10; observations on, 281; noticed by Hippocrates, 435; historical particulars of, xxxiii. 209; remarks illustrative of, 231; physiological observations on the causes of it, 433.

CRIBB, W. O. his account of a singular delivery, in which the vagina was imperforated, xiv. 17; observations on that case, by Mr. Dawson, 155; reply in explanation, 242; Mr. Cooper on the same case, 246; Dr. Crane on the case, 416.

CRICHTON, Dr. Alexander, his answer to Dr. Beddoes, ii 201; reply to, 308; his case of œdema fugax, vi. 26; case of undescribed jaundice, 29; reply to, 218; observations on the case, 312, 313; his synoptical table of diseases, xi. 379; on consumption, xx. 268.

————, Dr. of St. Petersburgh, his statement of the small-pox in Russia, xxx. 117; on the means of supplying vitality, xxxi. 431.

CRICHTON, Colonel Patrick, his invention of a bed for the conveyance of sick persons, xx. 142.

CRITICISM, observations on verbal, xxi. 89; animadversions on periodical, xxii. 441, 444.

CROCODILE, odorous scent of the eggs of, ii. 68; difference between that of the old and new world, vii. 575; account of a fossil one found in England, xv. 391; description of the, xvii. 485; one found in a fossil state in England, xxiv. 101; description of one in a fossilized state, with an engraving, xxv. 97; another discovered in Dorsetshire, xxviii. 516.

CROCUS, account of nine species of, xxiv. 439.

———— SATIVUS, soporific qualities of the, xx. 15; when introduced into medicine, xxviii. 320.

CROCHFORD, W. his cases of vaccination, xii. 207.

CROFT, Dr. his correction of an error concerning the cow-pox in his family, ii. 391; his evidence before the House of Commons on Dr. Jenner's petition, viii. 24.

CROSS, John, his sketches of the Medical Schools of Paris, xxxiv. 478.

CROTALUS HORRIDUS, odour of musk exhaled by, vi. 345; chemical examination of the poison of, xiv. 479; natural history of the, xxiv. 391; case of a man bitten by one, 394.

CROTCHET, on its use in premature labour, xi. 496; on a case requiring the use of the, xv. 1; caution respecting it, 3.

CROTOLARIA SALTIANA, a new plant from Abyssinia, described, xxvii. 347.

CROUP, virtue of the Seneka root in, i. 83, 503; inaugural dissertation on, 106; on the danger of the, 492; on the treatment of, ii. 146; contagious, 147; bleeding and blisters recommended in, 148; use of embrocations in, 149; two species of, iii. 56; bleeding in it condemned, 57; utility of leeches in, 240; objections to bleeding in, 540; successfully treated with digitalis, iv. 19; pathognomic symptoms of, 34; fatal cases of, 35; account of it as it appeared at Chesham, in Buckinghamshire, 67; efficacy of digitalis in, 421; on the symptoms and treatment of, v. 15; cases of, 516; on the occasional fatality of, ix. 89; causes of, 90; produced by a wound, x. 6; on the use of calomel in, xi. 227; symptoms of the, xiii. 555; on the proximate causes of it, 556; inutility of bleeding in, xiv. 184; cases of chronic, xv. 507; its analogy with hydrophobia, xix. 119; causes of it at Edinburgh, 184; success of calomel in, 215; xxi. 518; cases of chronic and spasmodic, 322; fatal in Edinburgh, xxiii. 172; on the severity of, 523; leeches recommended in, xxiv. 479; in an adult, xxvi. 377; observations on the case, 455; remedy for the, 509; on a fatal case of, xxvii. 44; observations on, 195; anatomy of the trachea in, 197; whether infectious, 200; common to maritime situations, 202; treatment of, 204; warm water, 206; bleeding, 207; calomel recommended in, 209; Seneka effectual in, 211; Dr. Mitchell's own case of, 213; success of mercury in, 282; on the distinction between spasmodic and inflammatory, 353; on Dr. Cullen's opinion of the, ibid.; on the nature of the disease, 354; accurate notions of Morgagni on, 355; membranes ejected from the trachea in, ibid.; on the substances called polypi in, 356; process of inflammation in, 357; opinions of Hunter on, ibid.; Bichat's description of, 358; on the membrane formed in the trachea, 359; on the effusion and coagulation of lymph, 360; fate of a patient dependent on the quantity of coagulum, ibid.; question of its being contagious, 362; use of the lancet in, 364; emetics serviceable in, ibid.; mercurial salts recommended in, 365; cases of, ibid.; case of, by Mr. Cornish, xxix. 268; effects of copious bleeding in, xxxi. 81; raucedo catarrhalis mistaken for, 358; hepar sulphuris, a remedy for, 518; analysis of a French treatise on the, xxxii. 47; diseases analogous to, observed in various animals, 50; on the diagnosis of the, 51; authors who have written on the, ibid.; on the sound of the voice in, 53; different species of, 54; on calomel in, 55; on bronchotomy as a relief in, 56; efficacy of calomel and moschus in, 80; account of an historical fragment in, xxxiii. 247; case of chronic, 265; observations on that case, 272; explanation concerning the same, 449.

CROW, Francis, his collection of fossils, xxvii. 28.

CROWFOOT, William, his statement of a case of apoplexy, vi. 549; answer to the statement, 552; explanation in reply to, vii. 77; his observations on Dr. Langslow's opinions, 89; remarks on his case of apoplexy, 138; Dr. Girdlestone on that case, 149; his explanation, 150; remarks on, 151; Dr. Langslow in answer to, 273; his vindication, 361; on the controversy excited by, 363, 397, 437, 444; on the cure of cataract, 404; Dr. Langslow's animadversions on, 456; his explanatory reply to Dr. Langslow, viii. 115; his case of burn treated with turpentine, xiii. 14; his two cases of carditis, xxii. 252; his case of amaurosis, xxxiii. 468; on the effects of poisoning with arsenic, xxxiv. 441.

CROWTHER, Bryan, his remarks on insanity, xxvii. 513.

————, Dr. case of induration of the vesiculæ seminales, prostate glands, and rectum, iv. 506; case of mania, 508; case of scorbutus, with dyspepsia, 510; case of the effects of opium, 512; his case of abscess in the abdominal muscles, xvi. 77; of syphilitic ulceration of the skin, ibid.

————, Messrs. their singular method of treating compound fractures, vii. 307.

CRUCE, Andrew, the inventor of the forceps for extracting the stone, ix. 19.

CRUICKSHANK, W. on the treatment of lues venerea, by nitric acid, i. 102; his remedy for diabetes, 145, 218; on the matter of ulcers, 433; death and character of, iv. 89; on the peculiar lymph of the absorbents, vi. 138; on the anastomosis, vii 139; on destroying the fætor of hospital sores, xxiii. 398.

————, William, (of Woolwich,) his experiments on galvanic electricity, iv. 126; vi. 211; Dr. Priestley in reply to, vii. 380; viii. 305, 307; his analysis of urine, ix. 351, 354; additional remarks on his experiments, x. 273.

CRUMP, H. his case of fistula lachrymalis, xi. 551.

CRUMPE, Dr. on the inefficacy of opiate frictions, vii. 125; remarks on, 346, 350, 355; viii. 327, 333, 346, 389, 393; on intestinal vermes, x. 550.

CRURAL ARCH, description of the, xi. 50; on its tendinous or ligamentous nature, ibid.; its ring larger in the female than the male, 51; on the hernia in the, 52; method of operating for it, 53; on the situation of the hernia with respect to the crural blood vessels, 54; situation of the structure in the incarcerated hernia, 55; on the means of removing the stricture, 56; directions for cutting the, 119; farther observations on the operation, 254.

CRUSADES, the means of introducing leprosy into Christendom, xxii. 409; advantages resulting from those expeditions, 411; establishments for the support of them, 413.

CRUSTA LACTEA, the viola tricolor a remedy for, xii. 224; xxi. 13; cases of, xv. 33; efficacy of blisters in, 34.

CRUSTACEOUS ANIMALS, yield a nutritious food, iii. 155.

CRUTCHES, improvement in the construction of, xxxiv. 128.

CRYPTOGAMIÆ, a new term proposed for that class of plants, xx. 16.

CRYSTALLINE LENS, analysis of the, xi. 165; on the absorption of the, xxx. 71; on discovering the proper capsule of the, xxxiv. 453.

CRYSTALLIZATION, on the varieties of, i. 73; observations on that of platina, iv. 373; on that of phosphoric acid, xi. 177; of essential oil of roses, 180; on the process of, xxix. 19.

CRYSTALS, observations on, iii. 88; on the rocky ones of Switzerland, iv. 469; on separating them from the beryl, vi. 473; obtained from gold by electricity, 475; examination of those of cinchonin, xxvii. 298.

CUBA, customs of the original inhabitants of, xxv. 480.

CUCUMBERS, the juice of them efficacious in consumption, ix. 24; description of fourteen species of, xxix. 434, 436.

CUFF, Mr. his evidence on Dr. Jenner's petition, ix. 66.

CULLEN, Dr. William, on sal æratus in calculous complaints, i. 296; his observations on petechiæ, 421; remarks on his theory, 486; on the stimulating principle, iii. 186; his opinion of punch and wine in fever, 217; doubted the existence of catalepsy, 402; his treatment of diabetes, v. 66; on his method of classification, 107; his definition of synocha, 234; his theory of fever, 388; comparison of his theory and practice, 388, 577; on the Portland powder for the gout, 419; on hæmorrhœa pete chialus, vi. 233; his observations on opium, vii. 127; on the tympany, 232; his controversy with Brown, 368; on emetics in apoplexy, 459; on the benefit of amusement, viii. 242; on the nervous fluid, 332; on white swellings, 493; on the contagion of catarrhal fever, ix. 527; remarks on his opinions, x. 536; xi. 79; anecdote of him, xi. 568; on his arrangement of diseases, 380; on the virtues of marsh trefoil, xii. 128; vindication of his opinions, xiii. 136; on the spasmodic nature of croup, 557; on rheumatism, 561; his opinion of chlorosis vindicated, xv. 142; on the virtues of oak bark, xvi. 69; on the external use of oil, 80; on his theory of rheumatism, 495; xviii. 429; recommends arum maculatum as a stimulant, xvii. 72; on his febrile theory, xviii. 76, 462; on the chin cough, 566; on the virtues of fumitory, xix. 72; on the benefit of broom in dropsy, 145; on diseases subsequent to measles; 147; on his system, 180; his opinions in pulmonary consumption, 266; on the properties of wormwood, 356; on the virtues of colt's-foot, 359; observations on his defects, xx. 79; on rheumatism, 357; objection to his theory of menstruation, xxi. 29, 30; on the sanguine and melancholy temperaments, 200; on the influence of the nervous power, 203; on gastritis, 276; his partiality to antimony, xxii. 41; on the sea-scurvy, xxiii. 234, 236; his errors on scrofula, xxiv. 149; on snuff-taking, 140; on his theory of gout, xxvi. 201; his opinion of case makers, xxvii. 103; on croup, 353; the study of his works recommended, xxix. 446; on his classes of phlegmasiæ, hœmorrhagiæ, and profluvia, xxx. 181; his observations on nurses, xxxi. 292; observation on his practice of bleeding and purging in fever, xxxii. 183; on cases of poison by opium, 273; on the effects of fumes of charcoal, 274; his doubts on pemphigus, 287.

CULLEN, Peter, on white serum of the blood, xxv. 300.

CULLURNE, J. his case of small-pox after vaccination, xii. 539.

CUMING, Dr. Ralph, his observations on old ulcers, vi. 156; on the influenza, x. 312; on the use of white vitriol in ague, 496; remarks on that paper, xi. 129; his reply, 248; his cases of sphacelus, 296; on gunshot wounds, 383; on the death of a child, 518; on typhus, xii. 52; reply to his observations on the death of a child, 73; on digitalis in dysentery, 113; on pulmonic inflammation, 114; on the vaccine prophylaxis, 209; on leprosy, 404; case of dysentery, 482; on nitre in sphacelus, 483; on fumigation in contagion, 484; his treatment of Dr. Scott censured, xiii 135; on his cases of sphacelus, 324; his reply, 325; on mercury in venereal affections, 395; his treatment of leeches, 448; his case of ischuria, 449; on the introduction of the catheter, 450; his practice commended, 549; his remarks on scrofula, xiv. 36; his vindication of naval surgeons, 37; on mercurial inunction in fevers, ibid.; reply to his observations, 46; on the use of cold water in scalds, 239; on the effects of the same in ophthalmia, 241; reply to him on cold water in scalds, 397; his treatment of leeches, xiv. 448; xv. 37; his case of ischuria, xiv. 448; on the introduction of the catheter, 449; xv. 39; remarks on his case of ischuria, and on the catheter, xv. 39; his case of tinea capitis, 43; his case of trismus, 44; his reply to Mr. Thomas, 224; on the use of nitre in sphacelus, 504; account of his treatise on fever, xvi. 570; on the refrigerant treatment of fever, xviii. 197; his remedy for gangrenes, 199.

CUPPING, supposed to be efficacious in yellow fever, ix. 99; advantageous in catarrhal peripneumony, x. 425; general benefits of the practice, 427; recommended for the opening of an abscess, xi. 327; advised by Hippocrates for pains in the head, xii. 470; its alleged utility in hydrophobia, 343, 527; xiii. 242; defects in the manner of performing the operation, xxii. 204; observations on the practice, 205; advice concerning it, 206; instructions for performing the operation, 376; farther directions, by another practitioner, 388; necessity of cautions in, 487; on obtaining the proper quantity of blood, ibid.; utility of the spirit lamp in, ibid.; improved scarificator for, xxiv. 177; an improvement in the practice, xxxii. 476; treatise on the nature of, xxxiv. 77.

CUPRUM AMMONIACUM, its efficacy in chorea, i. 22; beneficial in epilepsy, 23; the preparation of it, xiv. 46.

CURATIVE ART, account of a history of the, iii. 172.

CURCUMA, description of fourteen species of, xxix. 434, 436.

CUREAUDEAU, F.R. his account of an artificial stony formation, xxiv. 316.

CURRANTS, red and black, their medicinal virtues, xii. 225, 226.

CURREY, Dr. Gilbert, his case of tetanus cured by cold water, xxxi. 152.

CURRIE, Dr. James, his advice on the application of cold water in fever, i. 18; iii. 53, 55; on the virtue of nitric acid, i. 100; his description of medical thermometers, 304; value of his discovery, iv. 105; observations on his practice, 397; his obligations to early writers, 400; observations on his Life of Burns, v. 99; communications to him on the efficacy of cold affusion, vi. 381; his remarkable cure of tetanus, viii. 89; notice of his medical reports, ix. 8; on the influenza, x. 213; on cold water in convulsions, 410; success of his practice proved in the Mediterranean, xi. 22; character of, xv. 91; anecdotes of, 282; on the external use of oil, xvi. 80; not the first who applied cold water as a remedy for fever, xx. 82, 88, 164; observations on his directions, 562; his observations on the effects of cold in abating thirst, xxv. 473; on the effects of cold bathing on the system, xxvi. 399; his rules for the practice of attrition, 431; his theory and practice confirmed by facts, xxviii. 294; on the changes of the pulse, xxx. 190; his treatment of fever, xxxii. 183; on spunging the body with vinegar, xxxiii. 359.

CURRIE, Dr. of Chester, his account of the influenza, x. 213.

———, Dr. William, on the causes and cure of remitting bilious fevers, i. 105, 246; his memoirs of the yellow fever, 206; on the insalubrity of marshy situations, vi. 493; on the treatment of the malignant yellow fever at Philadelphia, ix. 97.

CURRY, Dr. James, elected physician to Guy's Hospital, vii. 384; on the use of mercury in hepatic complaints, xxii. 418; on the nature of the hepatic function, 421; his case of remitting ophthalmia cured by opium, xxx. 237; on the affinity of neuralgia and hemicrania, xxxi. 187.

CURTIS, William, his description of the mesembryanthemum pinnatifidicum, ii. 296.

CUSHION, in the foot of the horse described, xxiii. 343.

CUSTANCE, George, on singular cases of monstrosity, i. 451; on the cure of hœmorrhoids, iii. 334; his remarks on quackery, iv. 16; on yeast in typhus, 19; reply to, 231; on the fatality of croup, 420; on the efficacy of digitalis, 421; on the cow-pox, ibid; farther observations on yeast in typhus, v. 143; account of a contagious fever, 145; reply to, 270; animadversions on, 422; on an instrument for drawing teeth, vi. 251; his case of cancer, vii. 390; his letters on the advantage of the cool treatment of gout, ix. 124; on the influenza, x. 200; on vaccination, xi. 251; his case of vertigo, in which an insect was voided from the nose, 450; on the death of a child, xiv. 1.

CUTANEOUS ABSORPTION, observations on the nature of, iv. 9, 109; experiments illustrative of, xv. 274; not effected in the warm-bath, xxvi. 399; experiments and observations on, xxxi. 80.

——— AFFECTION, extraordinary instance of it in two brothers, viii. 508; suspends the vaccine operation, xiii. 417; affirmed to be a new principle in the origin of diseases, xxx. 228.

——— DISEASES, efficacy of hot flour applied externally in, i. 84, 152; enula helenium, a remedy in, 298; case of one, proceeding from cold, resembling scurvy, 420; effects of fish-diet in creating, iii. 68, 153, 252; benefit of a new species of salt in, 85; effects of oxygenated pomatum in, 167; case of pemphigus major, 552; benefit of the ledum palustra in, iv. 98; efficacy of sulphuric acid in, 482; benefits of the daphne mezereum in, v. 279; remarkable one in infants, 511; an inveterate one, occasioned by Jesuits' Drops, 519; extraordinary one in Norway, vi. 13; efficacy of olive oil in, 72, 248; case of lepra græcorum, 124; another of hæmorrhœa petechialis, 233; benefit of rhus radicans in, 272; general view of, 297; classification of, 298; particular descriptions, 300; effects of vaccination on, vii. 252; account of a topical remedy for, 293; observations on psora, 520; account of a singular one resembling small-pox, viii. 106; dangerous effects of calomel in, 113; on pustulous eruptions of the scalp, 200; extraordinary deformity in the cuticle, 508; effects of the oil of walnuts in, ix. 94; an unguent for, 271; on the leprosy in the middle ages, 555; efficacy of the Bath waters in, x. 183; instance of petechia without fever, x. 444; observations on febris urticaria, 481; effect of eating muscles in producing, 483; description of the pustules, 485; case of a boy whose skin was blue, 575; suroxygenated muriatic acid effectual in, 576; efficacy of the flowers of zinc in, xi. 93; general observations on, 230; characteristics of particular kinds of, 233; divisions and descriptions of, 234, 240; efficacy of the extract of hemlock in, 342; use of the juice of the water-flag in the cure of, 449; benefit of the common scabious in, 532; goose-grass beneficial in, 534; effects of the miryca in, xii. 37; on those which appear among the children of the poor, 97; on those connected with vaccination, 98; constitutions peculiarly liable to, 99; effects of the cynoglossum in, 125; marsh trefoil beneficial in, 128; powers of the nightshade in curing, 134, 135; effects of vaccination in removing, 194; peculiar one at Madeira, 195; benefit of the lesser centaury in, 221; remedy for those in children, 224; use of charcoal in, 283; mistakes concerning those ascribed to vaccination, 358; good effects of winter parsnip in, 365; hemlock dropwort, serviceable in, 366; on the cure of the leprosy, 404; remarkable one among sheep, 458, 461; benefit of pimpernella in, 456; disagreeable one of the head, xiii. 24; why they are prevalent in the Highlands, 35; causes of them among various nations, 36; on eruptions attending vaccination, 415; effects of the round-leaved sundew in, xiv. 64; benefit of convallaria in, 67; effects of curled-leaved dock in, 70; effects of arsenic in certain cases of, 113, 115; solution of arsenic successful in an eruption of the face, 116; general description and treatment of, 362; on those subsequent to the cow-pox, 378; efficacy of the viola tricolor in venereal ones, 432; description of a girl whose skin was blue, 471; observations on the causes of that appearance, 474; remarks on prurigo, 525; efficacy of mezereum in, 544; on crusta lactea, xv. 33; on lepra, 79; strange one among labourers in a coal-mine, 93, xvi. 472; benefit of stone peppercrop in, 266; common agrimony a remedy in, xv. 270; on the effects of Fowler's mineral solution in, 297; on the dangerous consequences of mistaking, 580; benefit of the external use of oil in preventing, xvi. 80; observations on three descriptions of, 178; on eruptions combined with cow-pox, 248; common celandine beneficial in, 559; benefit of arsenic in certain cases of, xvii. 253; case of lepra treated successfully with kali sulphuratum, 313; remarks on the case, 316; cases of pemphigus, xviii. 43, 44; antirrhinum a remedy for, 171; one peculiar to children, 344; carrot-poultice serviceable in, 349; good effects of fumitory in, xix. 72; benefit of dandelion in, 167; on the virtues of arsenic in, 206; efficacy of hypochœris maculata in, 257; remarkable case of enterodynia, 524; on the description and treatment of, xx. 273; on the urticaria, 274; varieties of the roseola, 278; on purpura, 280; on erythema, 281; on erysipelas, pemphigus, and pomphylox, 371; observations on phlegmon, 373; on the sweating sickness, 473; on hereditary ones, xxi. 230; singular case of one after vaccination, 250; on the several orders of, xxii. 109; historical account of the leprosy, 407; a pseudo syphilitic in Norway, 432; description of an eruptive disease on the integuments of the penis, xxiii. 441; affinity between that complaint and other diseases of the skin, 443; on the dangerous consequences of curing particular ones, xxiv. 122, 125, 214, 219; remarkable case of scabies, 162; observations on, 255; a remarkable one resembling cow-pox in South America, xxv. 241; produced by eating fish, 398; effectual remedies for, 498; the lacerta agilis, a remedy for, xxvi. 250; benefit of cinchona in, 335; case of erythema mercuriale, 341; benefit of the warm-bath in, 399; advantage of cold applications in, 433; observations upon one of the prepuce, 495; effects of tobacco in, xxvii. 45; peculiar to the Icelanders, 120; observations on erythema mercuriale, xxviii. 61; frequency of them in Ceylon, xxix. 163; but lessened there by vaccination, 164; case of purpura hæmorrhagia, 419; account of a practical synopsis of, 432; on the uses of arsenic in, xxx. 22; remarks on the operation of that medicine, 27; cases of the efficacy of arsenic in, 296; in psoriasis diffusa, 297; lepra vulgaris, ibid.; lepra alphos, 298; psoriasis scrotalis, 299; history of a fatal case of petechiæ without fever, 339; practical synopsis of, 412; description of the several forms of, 414; arrangement of the orders, 416; observations on lupus, 417; account of lepra, 418; benefit of the sulphur waters in, 421; efficacy of diluted alcohol, as a lotion in, 422; on the arsenical solution in, ibid.; benefit of the solanum dulcimara, as a decoction, in, ibid.; on the lepra nigricans, 423; fatal case of pemphigus, xxxi. 264; observations on that complaint, 268; account of the Mal d'Aleppo, 282, 372; case of partial perspiration, 392; history of a remarkable one at sea, 441; recipe of a new sulphurated soap for, xxxii. 79; case of universal eruption, 102; on the hereditary nature of, 143; the pemphigus in an epidemic state, 165; benefit of hepar sulphuris in, ibid.; observations on those which resemble syphilis, 230; historical researches on pemphigus, 231; case of leprosy, 355; identity of lepra and psoriasis, 356; case of psoriasis diffusa, 357; cases of pemphigus, with observations, 397; on insects as the cause of, 496; varieties of scabies, 498; etymology of the word scrofula, 499; case of a white man whose skin became black, xxxiii. 73; practical observations on porrigo and impetigo, 128, 136; case of exudation of the blood by the pores, 246; Chinese method of treating eruptive diseases, 248; case of supposed confluent varicella, 274; history of a tubercular eruption of a syphilitic appearance, 410; new method of treating the itch, 515; on the guinea worm, or malis dranunculus, xxxiv. 57; hints on the treatment of, 78; characteristics of those belonging to the true syphilis, 226; prevails epidemically in France, 171; efficacy of hepar sulphuris in, 257. *See Eruptions.*

CUTANEOUS PERSPIRATION, inquiry concerning it, and in the operation of sudorifics, i. 287.

CUTHBERTSON, WILLIAM, on distinguishing the two kinds of electricity, viii. 572; on galvanism, xx. 474.

CUVIER, M. his natural history of animals, i. 97, 208; on the colour and temperature of blood, 203; his account of a monstrous fœtus, ii. 480; on the hearing faculty of whales, 481; on the circulation of the blood in particular animals, iii. 376; on the nutrition of insects, ibid.; on the organs of voice in birds and other animals, vi. 279; observations on his galvanic experiments, 418; on the lines of the face, vii. 373; on the crocodiles of the ancient and modern world, 575; translation of his lectures on comparative anatomy, viii. 365, 466; on the iris of birds, xvii. 413; on the muscles connected with the organ of hearing, 388, 393; on the essence of vitality, xx. 108; on the structure of the spleen, 213; on the swimming bladder of fishes, xxiii. 454; on the medicinal leech, xxvi. 412; his observations on fossil remains, xxvii. 23; his scientific character, 25, *note*; his mineralogical inquiries in the neighbourhood of Paris, 27; account of his work on the fossil bones of quadrupeds, xxix. 520; his memoir on vomiting, xxx. 216; his observations on bats, 429; on his classification of animals, 431; on the comparison of bones, 432; his defence of Fourcroy, 504; denies circulation in insects, xxxi. 515; his report of the proceedings of the French Institute, xxxiii. 396; on the mouths of fishes, 398.

CYCAS, on the female flowers in that class of plants, xxx 254.

CYDER, beneficially used in sick head-ache, i. 287; description of the several kinds of, xvi. 170.

CYLINDER, description of one for facilitating evaporation, x. 479; improvement in the construction of the electrical, xx. 287.

CYMBIDUM COCCINEUM, description of the, xxvii. 260; account of another species, xxviii. 165.

CYNANCHE LARYNGEA, description of the complaint, v. 15; early bronchotomy necessary in, x. 6; case of, with an engraving, xxix. 125; cases of, xxx. 165; on the term, 166; morbid appearances in cases of, 237; a case of it, xxxii. 147.

——— MALIGNA, case of dysphagia succeeding, viii. 34; its resemblance to scarlatina anginosa, 496; epidemical, xxxii. 52; its inflammatory character, xxxiii. 97.

——— PAROTIDEA, or MUMPS: description of that complaint, v. 412; benefit of sal ammoniac and vinegar in, xiii. 509; an endemic one at sea, xx. 184.

——— TONSILLARIS, description of that disease, v. 15; unusual secretion of mucus in, ibid.; remarkable instance of, xiv. 150; benefit of oak-bark in, xv. 69; violent cases of, xix. 183; frequent at Edinburgh, 279; cases having a near resemblance to, 282; obstinate instances of it, 285; cured by blisters, xxi. 82; instances in which it was combined with catarrh, 259; removed by cathartics, 433, xxii. 257; fatal instances of it, xxii. 348; cases of it, xxiii. 524; instances of its severity, xxiv. 253.

——— TRACHEALIS, efficacy of the seneka-root in, i. 83, 106, 503; inaugural dissertation on the subject of, 106; on the danger of the, 492; on the causes of, 503; observations on the nature and treatment of, ii. 146; said to be contagious, 147; bleeding recommended in, 158; two species of the disease, iii. 56; bleeding censured in, 57; that practice vindicated, 240; use of opium and bleeding in, 539; objections to depletion in, 541; the death of General Washington, 473; observations on his case, 490; successfully treated with digitalis, iv. 19; different diseases classed under that name, 34; treated with emetics and bleeding, 35; most prevalent in a wet season, 36; account of its appearance at Chesham, 67; fatality of, 420; farther account of the efficacy of digitalis in, 421; remarks on the symptoms and treatment of, v. 15; history of cases of, 156; cases of, 516; remarkable circumstance in a case of, ix. 89; on its causes, 90; distinguished from acute asthma, ibid.; singular instance of, x. 6; on calomel in, xi. 227; observations on the symptoms of, xiii. 555; inefficacy of bleeding, xiv. 184; cases of chronic, xv. 507; infrequency of the complaint at Edinburgh, xix. 184; case of it, 214; obstinate instances of it, 322; governed by peculiarities of situation, xxi. 97; effects of calomel in, 518; fatal instances of, xxiii. 172; on the severity of the, 523; leeches and mercury recommended in, xxiv. 479; case of it in an adult, xxvi. 377; observations on its occurrence in adults, 455; remedy for the, 509; observations on the disorder, xxvii. 44; case of it in a man, 45; observations on, 195; anatomy of the trachea in, 197; general conclusions on the nature of, 198; accompanied with scarlatina, 199; communicated by contagion, 200; cases of it, 201; attended with inflammation, 203; on the proper treatment of, 204; bleeding beneficial in, 207; calomel and James'-powder efficacious in, 209; Dr. Mitchell's peculiar case of, 213; treated with mineral solution, 282; common in Christ's Hospital, xxxi. 482; on its connexion with measles, 483; sulphuret of potass serviceable in, 518; on diseases analogous to, xxxii. 47, 50; calomel successful in, 80.

CYNIPS ROSÆ, insects on the wild dog-rose, good for the tooth-ache, iv. 377.

CYNOGLOSSUM, or great hound's tongue, recommended in consumption, ii. 71; botanical description of, xii. 124; said to be deleterious, 125; classed with the aspinfoliæ, xxv. 123.

CYPRESS TREE, on its organs of fructification, xxx. 253.

CYRILLI, Dominique, his description of rare plants in Naples, i. 210; on mercurial applications in rheumatism, 385.

CYRUS, philosophy known to the Greeks in his reign, i. 437.

CYST, proportion of patients afflicted with dropsy of the single, viii. 388; on the cancerous, xv. 403.

CYSTIC OXIDE, a new species of urinary calculus, xxv. 254.

CYTISCUS MEANTHUS, a native plant of Hungary, xxvii. 260.

